# Boundary Extension in Face Processing

**DOI:** 10.1177/2041669517724808

**Published:** 2017-09-12

**Authors:** Olesya Blazhenkova

**Affiliations:** Faculty of Arts and Social Sciences, Sabancı University, Istanbul, Turkey

**Keywords:** boundary extension, false memory errors, face processing, object and spatial imagery, individual differences, emotion

## Abstract

Boundary extension is a common false memory error, in which people confidently remember seeing a wider angle view of the scene than was viewed. Previous research found that boundary extension is scene-specific and did not examine this phenomenon in nonscenes. The present research explored boundary extension in cropped face images. Participants completed either a short-term or a long-term condition of the task. During the encoding, they observed photographs of faces, cropped either in a forehead or in a chin area, and subsequently performed face recognition through a forced-choice selection. The recognition options represented different degrees of boundary extension and boundary restriction errors. Eye-tracking and performance data were collected. The results demonstrated boundary extension in both memory conditions. Furthermore, previous literature reported the asymmetry in amounts of expansion at different sides of an image. The present work provides the evidence of asymmetry in boundary extension. In the short-term condition, boundary extension errors were more pronounced for forehead, than for chin face areas. Finally, this research examined the relationships between the measures of boundary extension, imagery, and emotion. The results suggest that individual differences in emotional ability and object, but not spatial, imagery could be associated with boundary extension in face processing.

## Introduction

*Boundary extension* (BE) is a common false memory error, in which people confidently remember seeing a wider angle view of the scene than was actually viewed. They recall the surrounding regions of the scene, which were not visible during the encoding. BE is a constrained phenomenon, and scene extrapolation occurs just beyond the edges of a view (Gottesman & Intraub, 1999; Intraub & Richardson, 1989). This phenomenon is thought to comprise a two-stage process. First, a spontaneous extrapolation beyond the visible boundaries occurs during the initial encoding of an encountered scene. This happens due to a constructive nature of perception. It was suggested that the limited view of a scene activates a perceptual schema or a constructed visual-spatial representation of the expected layout outside the view (Chadwick, Mullally, & Maguire, 2013; Intraub, Bender, & Mangels, 1992; Intraub, Gottesman, & Bills, 1998). Subsequently, during the retrieval, BE manifests as a false memory error. In contrast to the traditional model of scene perception, which postulates that visual input is a single source of information in scene representation, the multisource model of scene perception proposes that scene perception involves processing in multiple modalities, one of which is vision (Intraub, 2010, 2012). According to the multisource model, scene perception is based on spatial egocentric framework. Multiple sources of input (e.g., visual sensory, amodal, conceptual, and contextual) ‘fill-in’ this framework with expectations about the surrounding visual field, which is only partially revealed in a picture. Thus, mental scene extrapolation does not happen after the stimulus is gone but already occurs during the scene presentation. In this view, BE is a source monitoring error (Johnson, Hashtroudi, & Lindsay, 1993) that arises due to the confusion between original sensory input and internally generated detail (Seamon, Schlegel, Hiester, Landau, & Blumenthal, 2002) and the attempts to determine which portion of the scene matches to the visual source. BE is considered to be a highly adaptive process that contributes to the representation of continuity and coherence of the world that exists beyond the limited visual input (Chadwick et al., 2013; Mullally, Intraub, & Maguire, 2012).

This phenomenon is remarkably robust. A variety of different methods, such as drawing from memory, recognition/rating of images that contain more or less of the original scene, or border-adjustment, consistently demonstrated BE effect (Hubbard, Hutchison, & Courtney, 2010; Intraub & Bodamer, 1993; Intraub, Hoffman, Wetherhold, & Stoehs, 2006). It was found in people of various ages, including 3- to 7-month infants and adults up to 84 years of age (Quinn & Intraub, 2007; Seamon et al., 2002). BE was observed for a wide range of target durations (250 ms to 15 s, Gottesman & Intraub, 2002; Intraub, Gottesman, Willey, & Zuk, 1996) and retention intervals (42 ms to 8 days, Intraub & Dickinson, 2008; Intraub & Richardson, 1989; Safer, Christianson, Autry, & Österlund, 1998). It is not limited to rectangular images but occurs for different shapes such as circular or irregular (Daniels & Intraub, 2006). BE is so robust that it is not eliminated even by the awareness of this phenomenon, though, test-informed instruction may lead to a reduction in BE (Gagnier, Dickinson, & Intraub, 2013; Intraub & Bodamer, 1993).

However, despite its robustness, BE does not occur for all kinds of image contents. It was found primarily for images containing a background surface, but not for images with depictions on blank backgrounds (Intraub et al., 1998). This was explained by the role of mental schema anticipating the continuous layout beyond the frame and not activating for images that do not depict partial view of a scene (Intraub et al., 1998). The hypothesis that BE occurs due to object completion was ruled out because this phenomenon was observed even for scenes that did not contain incomplete cropped objects (Intraub et al., 1992; Intraub & Bodamer, 1993). Besides, BE did not differ for scenes containing whole objects and cropped objects (Gagnier et al., 2013). BE was suggested to occur only for limited boundaries of the view, but not in response to isolated objects removed from the context background (Gottesman & Intraub, 2002, 2003). Though, there is contrary evidence that BE may occur for abstract scenes containing figures on a white blank background (McDunn, Siddiqui, & Brown, 2014). BE was suggested to be specific to representation of scenes or images that convey scene structure (Intraub et al., 1998). A scene is generally defined as a continuous spatial layout, which extends beyond the visible boundaries, while a nonscene refers to an isolated object, which is not embedded in a spatial context (Hubbard et al., 2010). More generally, a scene is defined as a depiction of a truncated view of a continuous world (Gottesman & Intraub, 2002). In this sense, cropped faces may be perceived, similarly to scenes, as truncated views of a full body extending beyond the edges of the perceived view (Intraub & Richardson, 1989).

Indeed, BE phenomenon was observed for a variety scene contents, including scenes with single or multiple objects, scenes with cropped and noncropped objects, and even abstract representations (Intraub & Bodamer, 1993; Intraub & Richardson, 1989; McDunn et al., 2014). However, the majority of studies did not focus on examination of BE in nonscenes. Possibly, BE may occur in response to some categories of nonscene objects, and in particular, to a very special kind of images, such as faces (Farah, Wilson, Drain, & Tanaka, 1998). Although human bodies and faces were sometimes included in the studied scenes (Gallagher, Balas, Matheny, & Sinha, 2005; Intraub et al., 2006; Ménétrier, Didierjean, & Vieillard, 2013; Munger & Multhaup, 2016), no research specifically focused on BE in face representations. There is some evidence that presence of humans and human faces may affect BE. For example, Gallagher et al. (2005) found an intriguing interaction between the background complexity and the presence of a person. When scenes contained a human, the amount of BE linearly increased with the complexity of the background, whereas for scenes without a human, the simplest and the most complex scenes yielded the highest amounts of BE. Overall, investigation of faces seems to be an overlooked dimension in the research on BE. Thus, the main purpose of the present study was to extend the existing knowledge on BE for nonscene images by examining this phenomenon in face processing.

Although a large body of evidence supports cognitive and neurological dissociation in processing *faces* and *scenes*, there is also research suggesting that processing of faces and scenes shares a lot of similarities. Functional magnetic resonance imaging research (Haxby et al., 2001; Kanwisher, McDermott, & Chun, 1997; McCarthy, Puce, Gore, & Allison, 1997) indicated that face processing is underpinned by the inferior occipital gyrus and lateral fusiform gyrus (also known as “Fusiform Face Area” or FFA). Lateral Occipital Cortex and FFA were found to have selective response to images of faces, but not those of houses or places (Kanwisher et al., 1997; Levy, Hasson, Avidan, Hendler, & Malach, 2001). In contrast, so-called Parahippocampal Place Area (PPA) was found to respond strongly to depictions of places, including indoor and outdoor scenes, and to respond weaker to buildings, but not to faces (Epstein & Kanwisher, 1998). Previously published research indicated the crucial role of hippocampus in scene viewing and processing spatial locations (Bird & Burgess, 2008). Neuropsychological literature documented cases of hippocampal damage followed by a selective impairment of scene recognition and intact face memory (Carlesimo, Fadda, Turriziani, Tomaiuolo, & Caltagirone, 2001; Taylor, Henson, & Graham, 2007). In addition, it was found that face processing relied on a detailed central scrutiny and was more strongly associated with processing of central information, whereas representations of buildings or scenes involved more peripheral information (Kanwisher, 2001; Levy et al., 2001). Neuroimaging research confirmed that BE is underpinned by the scene-selective regions of the brain.

Chadwick et al. (2013) indicated the crucial role of hippocampus in anticipation and construction of scenes, as well as in extrapolation of scenes beyond their physical borders. Research emphasized the role of hippocampus in construction of scenes both in memory and the imagination (Zeidman, Mullally, & Maguire, 2014). Mullally et al. (2012) found that patients with selective bilateral damage to hippocampus demonstrated less BE errors than control participants, supporting the role of hippocampus in BE. Inconsistent with this finding, Kim, Dede, Hopkins, and Squire (2015) found that amnestic patients with hippocampal damage exhibited BE similarly to healthy controls. Furthermore, Park, Intraub, Yi, Widders, and Chun (2007) demonstrated that BE task causes selective activation in the PPA, a region associated with processing scenes such as landscapes or buildings (Epstein & Kanwisher, 1998), but not in the Lateral Occipital Cortex, typically associated with object recognition (Grill-Spector et al., 1999).

Although many studies found the dissociation between face and place processing (Epstein & Kanwisher, 1998; Kanwisher et al., 1997; Levy et al., 2001; Taylor et al., 2007), there is also evidence that faces and places share neural underpinnings, for example, involvement of the ventral temporal cortex (Haxby et al., 2001). In addition, prosopagnosia (inability to recognize the faces) often co-occurs with topographical prosopagnosia (Landis, Cummings, Benson, & Palmer, 1986). Overall, given the earlier distinction between face and scene processing as well as the evidence of BE underpinnings by scene-selective brain areas, it could be possible that BE may not occur in response to faces. However, a cropped face image may elicit the sense of continuation beyond the picture boundaries (Intraub et al., 1992), similar to how a close-up portrait (disembodied head) may be interpreted as a continuous scene that includes the lower parts of the body (Intraub & Richardson, 1989). Possibly, a close-up face image may perform as a scene, and when being cropped, it may enforce the perception of continuity and mental reconstruction of a coherent representation in the same way as the truncated scene does. The present study aimed to examine whether *BE can be observed for cropped face images*. Particularly, to examine BE for faces rather than scenes, face images were presented on black backgrounds.

Furthermore, the present work examined whether there is an *asymmetry of BE* in face processing. Although BE typically occurs for all boundaries of an image, for example, four edges of a photograph (Intraub & Richardson, 1989), the asymmetry in BE, for example, different amounts of expansion at different sides of an image, was demonstrated by previous studies. In particular, BE was found to enlarge in the direction of the implied/anticipated motion, but not in the opposite direction (Courtney & Hubbard, 2004). Furthermore, a greater BE was found for the objects that typically move faster than for the objects that typically move slower (e.g., airplane vs. automobile). These findings may be explained by the role of BE in facilitation of the subsequent scene recognition (Dickinson & Intraub, 2008). For instance, anticipating a movement may increase BE in the expected direction and may aid spatial integration of the successive views (Hubbard et al., 2010). Similarly, implied motion in frozen-motion pictures and abrupt disappearance of a moving target cause displacement in memory toward the direction of motion (Freyd, 1983; Freyd & Finke, 1984; Futterweit & Beilin, 1994). Hubbard (1996) highlighted the similarity between BE and representational momentum, that is, displacement in memory of a moving target beyond the true final location. However, Munger, Owens, and Conway (2005) showed that BE and representational momentum are separate processes. They found that establishing the spatial layout occurs before the continuation of movement within a scene and concluded that BE cannot be due to a displacement in depth. Another evidence of asymmetry in BE comes from experiments using attentional cueing. As it was demonstrated by Intraub et al. (2006), BE can be affected by planned eye fixations: while BE appeared on the cued (to-be-fixated) side of the image, it was inhibited on the uncued side. Inconsistent with this finding, research demonstrated that focal and increased attention may constrain BE error (Dickinson & Intraub, 2009; Intraub, Daniel, Horowitz, & Wolfe, 2008). Overall, these findings implied that asymmetry in scene representation may be caused by the anticipatory processing in a certain direction.

The present work tested the hypothesis that *asymmetry in BE may exist for different facial parts*. This prediction was made based on the evidence for asymmetry in attention to different regions of a face. In particular, previous research demonstrated that upper face regions attract more attention than lower face parts (James, Huh, & Kim, 2010; McKelvie, 1976). Goldstein and Mackenberg (1966) examined the recognition of isolated portions of faces and demonstrated that upper portions of the face are more important for identification than lower portions. Most probably, this asymmetry in attention occurs because the eyes are the most salient and socially important part of the face (Janik, Wellens, Goldberg, & Dell’Osso, 1978). Our gaze naturally focuses on eyes and mouth face regions (Janik et al., 1978; Mertens, Siegmund, & Grusser, 1993; Walker-Smith, Gale, & Findlay, 1977; Yarbus, 1967), which are crucial for face identification and memorization and play important role in social communication (Ellis, 1975). Mouth region is also important but less salient than “eyes” region (McKelvie, 1976; Pellicano, Rhodes, & Peters, 2006). Markedly, the consistent looking preference for the upper part versus lower face part was not found for scenes (James et al., 2010). It was proposed that common configuration for faces determines top-focused pattern of exploration, whereas no such stereotypic patterns exist for scene exploration. Thus, due to asymmetry in attention in processing top versus lower facial parts, it was expected in the present research to find the corresponding top versus bottom asymmetry in BE of cropped face images. In addition, the present hypothesis about asymmetry of BE in face processing was inspired by the portrait composition “rules” from photography and visual arts, which put certain limitations on framing faces. There are common recommendations suggesting “good” places to crop and those to avoid. In particular, a crop is acceptable in a forehead area; however, it is advised to be avoided in a chin area. For example, according to Peterson (2016), the creator of Digital Photo Secrets website, “We are used to seeing pictures of people with the top of their head cropped off. It typically looks fine. The same cannot be said for the bottom of the face – do not remove someone’s chin!” The present study aimed to explore whether the location of a crop in face representation (forehead vs. chin) affects BE. Based on Intraub et al. (2006) findings, which indicate that BE can be amplified on the attended part of an image, it can be predicted that BE would be greater for the top (forehead) than for the lower (chin) part of the head. Alternatively, consistent with Intraub et al. (2008) study, which observed that focal attention may constrain BE, a greater attention to the upper part of the head may result in a reduction of BE. Gagnier et al. (2013) recorded oculomotor activity to study the mechanisms underlying the reduction in BE and to examine whether BE reflects a lack of eye fixations near the edges of a picture. They contrasted cropped and whole-objects stimuli and assumed that the cropped area creates a salient marker of boundary placement. The reduction in BE was expected at the side where the object was cropped with a picture boundary due to increased attention to the crop area. However, Gagnier et al. found that there was no difference in fixations to the boundary and to the cropped region. BE occurred in spite of multiple fixations to the boundary region and regardless whether the objects were cropped or not. In the present study, the oculomotor behavior was recorded to examine the distribution of attention. Consistent with findings of greater attention to upper face regions (James et al., 2010; McKelvie, 1976), it was expected to observe a higher oculomotor activity in the top part of the face, including eyes and cropped boundary regions. In addition, the present study explored the possible asymmetry in BE for lower versus upper parts of the face.

Finally, the present research intended to explore face *BE in relation to imagery*. Mental imagery seems to have a special importance in understanding mechanisms of BE. It was suggested that scene continuity activates schematic expectations representing the visual world beyond the picture boundaries, and such mental schema underlies not only perception and memory but also imagination of scenes (Intraub, 1997). Intraub et al. (1998) proposed that BE may occur regardless whether perception or imagination activated the perceptual schema. Indeed, a great body of evidence demonstrated the similarity between perception and imagery, which share common representations, cognitive mechanisms, and neural underpinnings (Farah, 1988; Ganis, Thompson, & Kosslyn, 2004; Kosslyn et al., 1999; Shepard & Metzler, 1971). In particular, O'Craven and Kanwisher (2000) found that imagery of faces and places activated the same stimulus-specific brain regions (FFA and PPA) as in perception, but the magnitude of activation was lower for imagery than for perception. Although visual imagery and perception showed significant overlap, research highlighted that imagery involves more top-down processing and more prefrontal cortex involvement than perception (Ganis et al., 2004; Mechelli, Price, Friston, & Ishai, 2004). Hubbard et al. (2010) showed that early visual processing areas were not involved in BE and proposed that BE is underpinned by high-level processes. These findings highlight the possible role of mental imagery in BE. However, previous studies provided only limited evidence about the relationship between imagery and BE. In particular, research examined how *imagery instructions* may influence BE. Although BE was found primarily for images containing a background surface but not for those with blank backgrounds (Intraub et al., 1998), imagery instructions altered these findings. In particular, BE was observed when participants were deliberately imagining extended backgrounds around objects on blank backgrounds (Gottesman & Intraub, 2002). Imagery may make people believe that they have experienced some events that they have not experienced (Loftus, 2003). Yet, it was proposed that the act of imagination per se does not always increase source memory errors. Foley, Foy, Schlemmer, and Belser-Ehrlich (2010) demonstrated that, in contrast to spontaneous imagery instructions, deliberate imagery instructions may even decrease source memory errors. Foley et al. claimed that the awareness about the act of generating the images might distinguish these imagined items from the actually seen items, thus leading to reduced source memory errors. Using a variety of imagery instructions, Munger and Multhaup (2016) found that explicit imagination of sensory details in the scene does not result in increased BE. They concluded that there is no imagination effect on BE.

While the majority of studies on imagery and BE primarily focused on the effect of imagery instructions on BE, there were only few attempts to explore the relationship between BE and *individual differences in imagery.* Previous literature reported individual differences in visual-object (visualizing pictorial appearances in terms of shape, color, and texture) and visual-spatial (visualizing spatial relations and transformations) imagery (Kozhevnikov & Blazhenkova, 2013; Kozhevnikov, Kosslyn, & Shephard, 2005). In particular, Kozhevnikov et al. distinguished between two types of individuals: object and spatial visualizers. Object visualizers tend to experience vivid and colorful mental images and to excel in object visualization tasks (e.g., recognizing degraded objects), whereas spatial visualizers tend to use imagery for representing spatial relations and transformations and to excel in tasks that require spatial visualization (e.g., mental rotation). This distinction in individual differences in imagery was based on evidence of the dissociation between ventral “visual-object” and dorsal “visual-spatial” pathways in the brain, underpinning processing of different aspects of visual information (Farah, Hammond, Levine, & Calvanio, 1988; Kosslyn & Koenig, 1992; Mazard, Tzourio-Mazoyer, Crivello, Mazoyer, & Mellet, 2004; Ungerleider & Haxby, 1994). The relationship between BE and individual differences in object versus spatial visual imagery was examined by Munger and Multhaup (2016). Using the Object-Spatial Imagery Questionnaire (OSIQ), assessing object and spatial imagery (Blajenkova, Kozhevnikov, & Motes, 2006), Munger and Multhaup found a significant positive correlation between *spatial imagery and BE,* but not between object imagery and BE. The authors concluded that BE might be related to a superior visual-spatial rather than visual-object imagery ability. In addition, there is evidence that individual differences in spatial but not object imagery are related to spatial dispersion of eye movements during the recall of scenes (Johansson, Holsanova, & Holmqvist, 2011). Furthermore, Mullally et al. (2012) asked participants to visualize in their imagination the scenes extending beyond the current view and to rate the vividness of their subjective imagery. Researchers found that patients with selective bilateral hippocampal lesions had significant impairments in the ability to visually imagine spatially coherent scenes (e.g., spatial relationships and locations). Markedly, the same patients demonstrated attenuated BE (thus, better memory) than control participants. These findings indicate that the subjective vividness of scene imagery may be associated with the increased BE. It is important to note that Mullally et al. (2012) measured vividness associated with imagination of spatially coherent scenes. As reported in Blazhenkova (2016), vividness that refers to imagery of spatial properties (locations, spatial structure, and relationships) versus pictorial object properties (color, texture, and shape) constitute separate, spatial and object, vividness dimensions. Thus, the results of Mullally et al. (2012) may be interpreted as a finding of the relationship between *spatial* imagery and BE for scenes.

At the same time, the existing evidence suggests the possibility of positive association between *object imagery and BE*. While some studies demonstrated that vivid and pictorial object imagery may facilitate memory (Marks, 1973; Vannucci, Pelagatti, Chiorri, & Mazzoni, 2016), other literature showed that the elaboration of sensory details during the encoding leads to the increase in source memory errors (Thomas, Bulevich, & Loftus, 2003). Research has suggested that vivid and rich in sensory detail imagery experiences may lead to later confusion between real and imagined experiences, causing false memory errors (Gonsalves & Paller, 2000; Gonsalves et al., 2004). Markham and Hynes (1993) found the relationship between individual vividness of imagery and memory errors in a task that, during the encoding, required to imagine half-shapes as complete symmetrical geometric forms. The participants were divided in “high-imagery” and “low-imagery” groups, according to their scores on Vividness of Visual Imagery Questionnaire (VVIQ; Marks, 1973). During the recall of shapes, high-imagery participants made more reality monitoring errors, confusing half-shapes with complete shapes, than low-imagery participants. Notably, in contrast to Mullally et al. (2012) assessing spatial vividness, the VVIQ instrument (Marks, 1973) measures object vividness (Blajenkova et al., 2006; Blazhenkova, 2016). Therefore, the results of Markham and Hynes may be interpreted as a finding of the relationship between object imagery vividness and mental extrapolation errors for objects (symmetrical shapes), but not for scenes. Besides, numerous reports provided evidence that higher VVIQ scores were associated with higher reality monitoring errors and false memory (Dobson & Markham, 1993; Gonsalves et al., 2004; Hyman & Pentland, 1996; Mazzoni & Memon, 2003). Overall, these findings suggest that vividness and strength of individual imagery may lead to increased BE errors. However, it is not yet known how BE may be affected by the type of imagery (object vs. spatial) and the content of the image (a single object on a blank background vs. scene). The present work examined the relationship between BE and individual differences in the two types of imagery: object versus spatial. The present investigation implemented nonspatial stimuli: faces on blank backgrounds. On the basis of the previous literature, it was expected that larger BE in face processing would be associated with superior object, but not spatial, visual imagery.

In addition, because the present research used face stimuli conveying emotions, the measures of *emotional processing* were also included along with the imagery assessments. Previous studies indicated that, similar to vividness, emotional content might induce the creation of false memories (Hyman & Pentland, 1996; Porter, Spencer, & Birt, 2003). However, the evidence regarding the relationship between emotional processing and BE is quite controversial. Several studies suggested that BE effect may depend on the emotional content of scenes. For example, Ménétrier et al. (2013) demonstrated that positively valenced stimuli (i.e., actors showing happiness and pleasure through facial and postural expressions) led to BE effect, whereas negatively valenced stimuli (i.e., expressing anger and irritation) did not produce directional memory distortion. Safer et al. (1998) showed that negative emotional content of an image may lead to “tunnel memory effect” or boundary restriction (BR), opposite to BE effect. However, other research found no difference in magnitude of BE for pictures with emotionally neutral and emotionally charged content (Candel, Merckelbach, Houben, & Vandyck, 2004; Candel, Merckelbach, & Zandbergen, 2003). Mathews and Mackintosh (2004) found that scene extrapolation interacts with individual differences in emotionality: BE for very negative scenes was reduced in high-trait-anxious individuals. Blazhenkova and Kozhevnikov (2010) indicated positive association between individual differences in emotion and object, but not spatial, imagery. Therefore, in the present study, it was expected that both, individual differences in emotional ability and object imagery, similar to each other, would be positively related to BE in face processing.

To summarize, there is an increasing body of research on BE in scene perception and memory. However, there are gaps in the knowledge regarding BE for nonscene representations, and in particular, face images. The present work examined BE errors in face processing using cropped face images on black backgrounds. Study 1 explored BE in different facial parts. Using short-term and long-term memory conditions, it examined whether the location of a crop (forehead vs. chin) affects BE. Study 2 examined the relationships between the measures of BE in face images and different assessments of individual differences in object/spatial imagery and emotion.

## Study 1

The goal of Study 1 was to examine BE using face stimuli, cropped either in a forehead or chin. The task was administered in two memory conditions to explore the robustness and the possible differences in BE effect. On the basis of previous research that showed BE for both long-term and short-term retention (Intraub & Dickinson, 2008; Safer et al., 1998), it was expected to find this effect in both memory conditions. The early onset of BE indicates that this phenomenon occurs at the border between perceiving and remembering (Intraub & Dickinson, 2008; Roediger, 1996). The comparison between the long-term and short-term memory conditions could elucidate the role of perception and memory in BE. Furthermore, it was hypothesized that participants would make BE errors for both crop locations. However, based on the attentional asymmetry in face processing, forehead extension was expected to be different from chin extension. In addition, this study explored the effect of the expressed emotion on scene extrapolation. Eye-tracking data were collected alongside with the task performance data.

### Method

#### Participants

Eighty-three^1^ participants were Sabancı University students (19–26 years old, *M* age = 22). Participants were run either in the short-term memory (22 males, 19 females) or in the long-term (19 males, 23 females) condition. They were reimbursed with course credits for their participation. The research was approved by the Sabanci University Research Ethics Council. All participants provided written informed consent.

#### Materials and procedure

All participants were tested individually. They completed either a short-term or a long-term condition of the *Faces Task*. This task included Encoding & Emotional Identification and Recognition parts. During the Encoding, participants observed photographs of faces, cropped either in a forehead or in a chin area. The stimuli were created based on Karolinska Directed Emotional Faces picture set (Lundqvist, Flykt, & Öhman, 1998). They comprised color pictures of female faces displaying six different emotional states (anger, disgust, happiness, neutrality, sadness, and surprise) photographed from the front. Each emotional state was presented in four different faces (two cropped in the forehead and two in the chin area), thus making 24 pictures in total. The same Encoding face stimuli were used in both conditions. Forehead- and chin-cropped faces had fixed width, but varied in height. The height of full uncropped original Karolinska Directed Emotional Faces images corresponded to the full height of the screen. The cropped stimuli were shifted so that the bottom of forehead-cropped faces touched the bottom of the screen, and the top of chin-cropped faces touched the top of the screen, thus leaving some space for a mental continuation of a cropped part. Each face appeared for 4 s, and it was followed by Emotional Identification.

The instruction included two parts. The Encoding & Emotional Identification instruction was as follows: You will see 24 pictures of people with different facial expressions. Try to identify the emotions conveyed in each of these photos. After each face presentation, you will be automatically taken to the page with a question about this face emotion. During the Emotional Identification, participants were asked, “What was the emotion expressed by this face?” They had to select among the seven answer options: “fear,” “anger,” “disgust,” “happiness,” “neutral,” “sadness,” and “surprise.” The answer time was not limited, and program proceeded to the next trial after the response. The recognition of the emotional expressions was included in the Faces Task to encourage participants focusing on the other face properties rather than local areas of crops and to separate the presentation of the Encoding and Recognition face stimuli.

The second part of the instruction was as follows: You will see the same picture again among the other options. Try to recognize the picture that you saw before. Select this picture by clicking your mouse on it. During the Recognition, participants had to recognize the previously seen images and to make a forced-choice selection among the four options. During the first trial of the Emotional Identification task, to ensure that a participant understands the task, the experimenter noted and showed that, though, all the choice images represent the same face, but the crop area is different. These selection options included the same faces with two extended and two reduced crops, representing different degrees of possible BE and BR errors. To increase the sensitivity of the test, none of the answer options were the same as an image presented during the Encoding (so there was no correct answer option). Some other BE studies also did not include the selection option identical to the studied view (Mathews & Mackintosh, 2004; Quinn & Intraub, 2007). For example, Mathews and Mackintosh (2004) used a forced-choice recognition task that presented four alternatives with different degrees of BE and restriction but did not present the originally memorized scene. Remarkably, research on eyewitness memories demonstrated that participants confidently and falsely identified someone in the lineup of potential suspects as being the perpetrator, when the actual perpetrator was not in the lineup (Wells et al., 1998).

**Figure 1.**
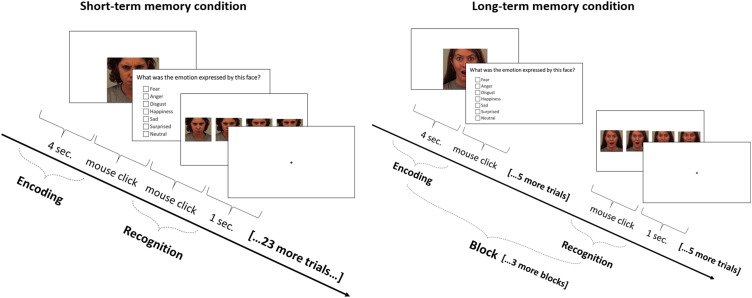
Schematic of trial and block sequences in the short-term and long-term conditions of the Faces Task.

In the short-term memory condition, both parts of the instruction were presented prior to start of the test. In the long-term condition, the second part of the instruction came after each block of Encoding & Emotional Identification. In the short-term memory condition, each Encoding image was immediately followed by the Emotional Identification and then Recognition (Figure 1). The response time for Emotional Identification was not limited and not recorded. Pilot participants testing showed that it took approximately 3 to 10 s. Because the time interval between the stimuli Encoding and Recognition did not exceed 30 s, this condition was labeled as “short-term” memory (Baddeley & Hitch, 1974). In the long-term memory condition, the encoding images were presented in four blocks, each containing six trials. Each Encoding block was followed by a Recognition block. This condition required long-term memory because the time interval between the Encoding and Recognition stimuli exceeded 30 s (Atkinson & Shiffrin, 1968; Baddeley & Warrington, 1970).

The same Recognition alternatives were used in both conditions. However, in the short-memory condition, half of the Recognition options increased in size from right to left, reflecting the magnitude of BE/BR in the solution options (i.e., Large BR, Small BR, Small BE, Large BE), and another half had the reversed order. In the long-memory condition, all choice options increased in size from right to left. To have a visual diversity in the selection options, the size of the face crops varied, and it was balanced between the chin and forehead areas. The face width was constant between the four alternative options. However, because faces were cropped differently in either forehead or chin, their heights were different. The order of face images with varying crop locations was intermixed. It was fixed for the Encoding and Recognition, which allowed to keep the time between the corresponding encoding and Recognition stimuli for the same face relatively constant. Behavioral responses (mouse clicks on the selected pictures) were recorded.^2^ The response time for Recognition was not limited. Eye-tracking data were collected using Tobii TX300 Eye Tracker (data rate 120, framerate 5, fixation filter I-VT) and Tobbi Studio software. Stimuli were presented full-screen on a 23” monitor at a resolution of 1,920 × 1,080 pixels.

### Results - Behavioral Data

#### Types of errors during the Recognition

**Figure 2.**
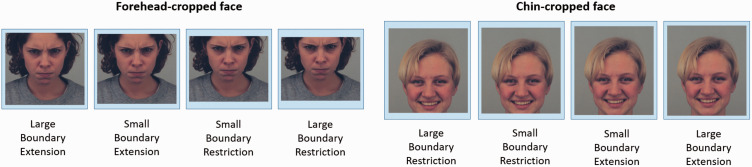
ROIs around different types of Recognition errors in the Faces Task. ROIs = Regions of Interest.

For the Recognition data analysis, Regions of Interest (ROIs) were created around each answer option (Figure 2). Because none of the answer options represented a correct answer, the Recognition was analyzed in terms of different types of errors (i.e., Large BE, Small BE, Small BR, and Large BR), corresponding to the four answer options. *Error Frequency* was the number of mouse clicks on different Recognition options. The two types of BE answers and the two types of BR answers were obtained in the forced-choice task, leading to two categories of responses: Extension and Restriction Errors. The mean frequencies of responses falling in these two categories were further compared for faces cropped in the forehead or chin areas.

#### Recognition analysis

**Figure 3.**
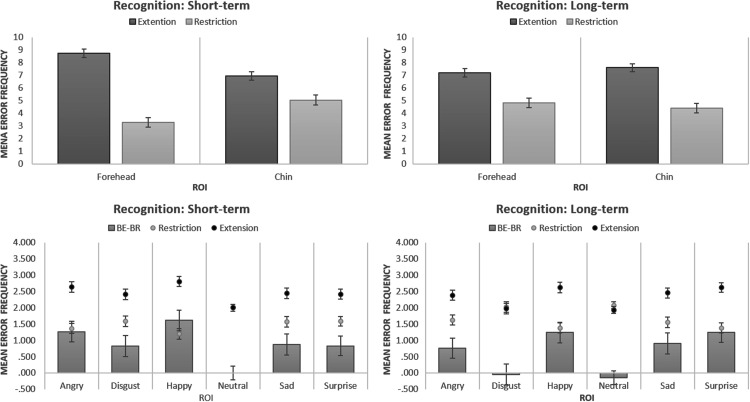
Recognition data: Error Frequencies for different types of errors in the Faces Task. ROI = Region of Interest; BE = Boundary Extension; BR = Boundary Restriction.

A mixed 2 × 2 × 2 three-way (two within- and one between-subjects factors) analysis of variance (ANOVA) was conducted to assess the impact of two repeated measures variables, Error Type (BE, BR) and cropping Location (Forehead, Chin), on participants’ recognition (Error Frequency), across the two memory Conditions (Short-Term, Long-Term).^3^ The adjustment for multiple comparisons was done with Bonferroni correction. The analysis revealed a considerable main effect for Error Type, *F*(1, 81) = 71.757, *p* < .001, partial η^2 ^= .470, showing that participants selected more extension (*M* = 7.617, *SE* = 0.191) and less restriction (*M* = 4.383, *SE* = 0.191) answer options. There was a significant but weak interaction between Error Type and Location, *F*(1, 81) = 4.562, *p* = .036, partial η^2 ^= .053, suggesting that Error Type effect differed depending on the cropping Location. While the extension effect was present for both locations of crop, it was greater for forehead-cropped images (*MD_BE-BR_* = 3.922, *SE* = 0.464, *p* < .001; *M*_*BE*_ = 7.961, *SE* = 0.232; *M*_*BR*_ = 4.039, *SE* = 0.232) than for chin-cropped (*MD_BE-BR_* = 2.546, *SE* = 0.532, *p* < .001; *M*_*BE*_ = 7.273, *SE* = 0.266; *M*_*BR*_ = 4.727, *SE* = 0.266) images. There was no interaction between Error Type and Condition, *F*(1, 81) = 1.380, *p* = .243, partial η^2 ^= .017, suggesting no difference in the Error Type effect between the short-term and long-term conditions. There was a weak but significant Error Type × Location × Condition interaction, *F*(1, 81) = 11.511, *p* = .001, partial η^2 ^= .124. A separate analysis for two conditions revealed that forehead versus chin asymmetry of BE effect was present in short-term condition, *F*(1, 40) = 15.426, *p* < .001, partial η^2 ^= .278, but not in the long-term condition, *F*(1, 41) = .783, *p* = .381, partial η^2 ^= .019. Figure 3 presents mean Error Frequencies of BE and BR responses for both conditions.

#### The effect of Emotion

The possible effect of Emotion on boundary extrapolation in cropped face images was additionally explored. A mixed 6 × 2 × 2 three-way ANOVA was conducted to assess the impact of Emotion (Anger, Disgust, Happiness, Neural, Sadness, Surprise) and Error Type on Error Frequency, across the two memory Conditions.^4^ The adjustment for multiple comparisons was done with Bonferroni correction. The analysis revealed a main effect of Error Type, *F*(1, 81) = 41.819, *p* < .001, partial η^2 ^= .340, demonstrating BE effect. There was no interaction between Error Type and Condition, *F*(1, 81) = 1.019, *p* = .316, partial η^2 ^= .012. There was a weak but significant interaction between Emotion and Error Type, *F*(5, 405) = 7.895, *p* < .001, partial η^2 ^= .089, suggesting that Error Type effect depended on the Emotion. This interaction effect was analyzed using a simple main effects analysis. Pairwise comparisons of the Emotion × Error Type interaction revealed that Mean Difference (BE-BR) was significant only for “Angry” (*MD_BE-BR_* = 1.015, *SE* = 0.220, *p* < .001); “Happy” (*MD_BE-BR_* = 1.424, *SE* = 0.221, *p* < .001); “Sad” (*MD_BE-BR_* = .891, *SE* = 0.227, *p* < .001); and “Surprise” (*MD_BE-BR_* = 1.034, *SE* = 0.212, *p* < .001) emotions. Furthermore, to compare the size of BE effect for different types of emotions, the additional repeated measures analysis with BE-BR as a dependent measure and Emotion as a within-subjects variable. Pairwise comparisons revealed that BE-BR difference for “Happy” emotion was significantly larger compared with BE-BR for “Disgust” (*MD* = 1.036, *SE* = 0.298, *p* = .012) and “Neutral” (*MD* = 1.494, *SE* = 0.261, *p* < .001) emotions. BE-BR discrepancy was significantly smaller for “Neutral” emotion compared with BE-BR for “Angry” (*MD* = −1.084, *SE* = 0.262, *p* = .001), “Sad” (*MD* = −.964, *SE* = 0.241, *p* = .002), and “Surprise” (*MD* = −1.108, *SE* = 0.273, *p* = .002) emotions. Figure 3 shows mean Error Frequencies of BE and BR answers for different types of emotions. In addition, for a clearer demonstration of Error Type effect for each emotion, this figure displays mean difference between BE and BR measures (BE-BR). There was no interaction between Emotion, Error Type, and Condition, *F*(5, 405) = 1.381, *p* = .231, partial η^2 ^= .017.

### Results - Eye-Tracking Data

#### Encoding analysis

**Figure 4.**
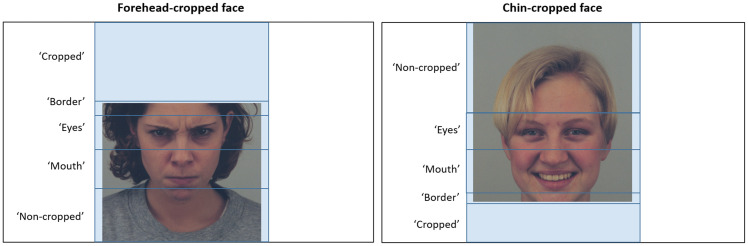
ROIs around different parts of the face viewed during the Encoding of the Faces Task. ROIs = Regions of Interest.

To analyze the visual processing during the Encoding, five ROIs were created for each cropped face image: “cropped” area, “border” area, “eyes” area, “mouth” area, and “noncropped” face area. As illustrated on the Figure 4, the “cropped” ROI was defined as a missing part of a face, whereas “noncropped” ROI was the area above the “eyes” in chin-cropped images or the area below the “mouth” the forehead-cropped faces. The “eyes” ROI was created around the eyes, between the top of the alar nasal sulcus and just above the superciliary arch. The “mouth” ROI included the area below the “eyes” ROI and mentolabial sulcus. The “border” ROI included the area between the “eyes” and “cropped” ROIs in the forehead-cropped faces or area between the “mouth” and “cropped” ROIs in the in chin-cropped images. Two different eye-tracking metrics, based on the ROIs defined in Figure 4, were used in the analysis. *Visit Duration* was the total time in seconds spent within a particular ROI. *Visit Count* was the total number of visits to an ROI, where visit is defined as a time interval between the first fixation inside the ROI and the next fixation outside the ROI.

**Figure 5.**
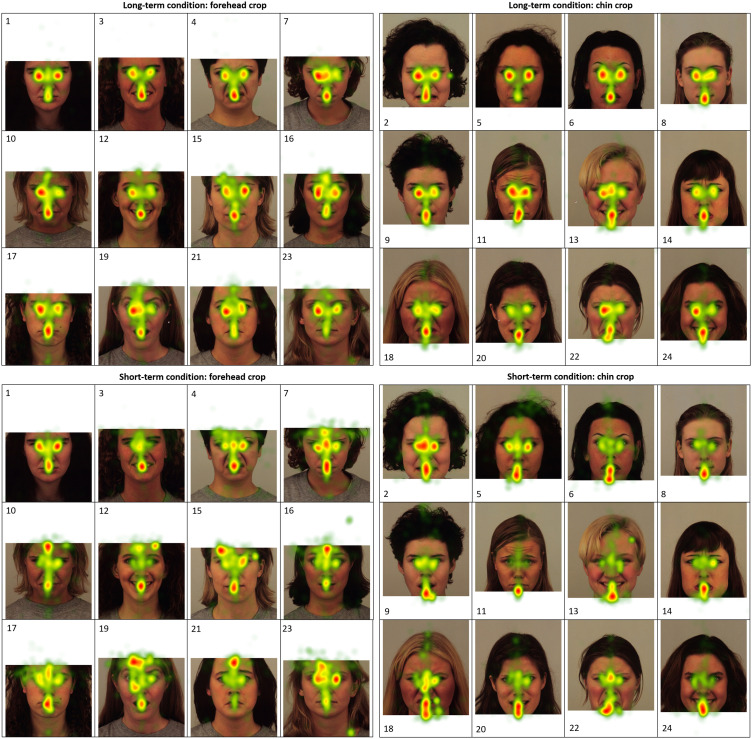
Encoding eye-tracking heatmaps representing relative Visit Duration in the short-term and long-term conditions of the Faces Task.

A mixed 5 × 2 × 2 three-way (two within- and one between-subjects factors) ANOVA was conducted to assess the impact of two repeated measures variables, Face Area (“cropped,” “border,” “eyes,” “mouth,” “noncropped”) and cropping Location (Forehead, Chin), on oculomotor variables (Visit Duration, Visit Count) across the two Conditions (Short-Term, Long-Term). The adjustment for multiple comparisons was done with Bonferroni correction. The data were analyzed separately for Visit Duration and Visit Count. Eye-tracking heat maps representing relative visit durations during the Encoding are presented in Figure 5.

**Figure 6.**
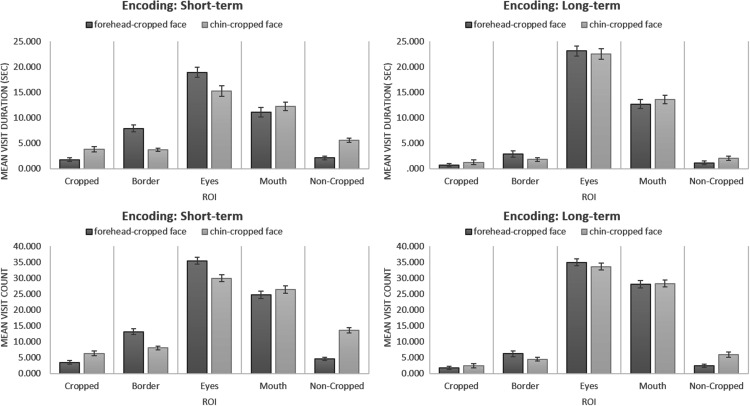
Faces Task Encoding data: Visit Duration and Visit Count for the different ROIs. ROIs = Regions of Interest.

The analysis of *Visit Duration* revealed a substantial main effect for Face Area, *F*(4, 324) = 259.601, *p* < .001, partial η^2 ^= .762. Pairwise comparisons demonstrated that “eyes” (*M* = 19.965, *SE* = 0.694) attracted significantly more attention than any other face areas all *p*’s < .001). The “mouth” was the second most salient area; mean Visit Duration was significantly greater for the “mouth” ROIs (*M* = 12.407, *SE* = 0.582) than for other ROIs (all *p*’s < .001), except for the “eyes” areas. Visit Duration for “cropped” (*M* = 1.880, *SE* = 0.227) and for “noncropped” (*M* = 2.736, *SE* = 0.226) areas were significantly smaller than all other ROIs (all *p*’s < .001), but not different from each other (*p* = .087). Mean Visit Duration for the “border” areas (*M* = 4.065, *SE* = 0.318) was greater than for the “noncropped” ROIs (*p* = .005). The effect of cropping Location was nonsignificant, *F*(1, 81) = 1.705, *p* = .195, partial η^2 ^= .021; there were no difference in Visit Duration between forehead- and chin-cropped images. The effect of Condition was not significant, *F*(1, 81) = .094, *p* = .760, partial η^2 ^= .001. There was a significant interaction between Face Area and cropping Location, *F*(4, 324) = 29.360, *p* < .001, partial η^2 ^= .266, a significant but weak interaction between Face Area and Condition, *F*(4, 324) = 14.886, *p* < .001, partial η^2 ^= .155, as well as a significant but weak interaction between Location and Condition, *F*(1, 81) = 18.751, *p* < .001, partial η^2 ^= .188. Mean Visit Duration for the “cropped,” “border,” and “uncropped” areas was greater in the short-term condition than in the long-term condition, but it was greater for the “eyes” area in the long-term condition than in the short-term condition (all *p*’s < .001). Face Area × Location × Condition interaction was also significant, *F*(4, 324) = 10.416, *p* < .001, partial η^2 ^= .114, indicating that the discrepancy in Face Area effect between the forehead- and chin-cropped images was different in short and long-term conditions. In both conditions, there were no differences in mean Visit Duration between the forehead versus chin areas for the “cropped” and the “mouth” ROIs. For the “border” and the “eyes” ROIs’, Visit Duration was greater for forehead- than for chin-cropped faces in the short-term condition (*p*’s < .001), but not in the long-term condition. Mean Visit Duration for the “noncropped” ROIs’ was greater for the chin- than for the forehead-cropped faces in the short-term condition (*p* < .001), but not in the long-term condition. Figure 6 demonstrates the results of comparisons between mean Visit Duration for different ROIs in the forehead- and the chin-cropped faces, separately for the two conditions.

Consistent with Visit Duration, the analysis of *Visit Count* revealed a substantial main effect for Face Area, *F*(4, 324) = 746.494, *p* < .001, partial η^2 ^= .902. Pairwise comparisons demonstrated that the “eyes” (*M* = 33.497, *SE* = 0.714) attracted significantly more attention than any other face areas all *p*’s < .001. The “Mouth” was the second most salient area; mean Visit Count was significantly greater for the “mouth” ROIs (*M* = 27.132, *SE* = 0.758) than for the other ROIs (all *p*’s < .001), except for the “eyes” areas. Mean Visit Count for the “cropped” areas (*M* = 3.481, *SE* = 0.325) was the smallest compared with all other ROIs (all *p*’s < .001). There was no difference between mean Visit Count for the “border” (*M* = 7.948, *SE* = 0.461) and the “noncropped” (*M* = 6.614, *SE* = 0.396) ROIs (*p* = .155). The effect of cropping Location was nonsignificant, *F*(1, 81) = 3.838, *p* = .054, partial η^2 ^= .045. The effect of Condition was significant but weak, *F*(1, 81) = 6.136, *p* = .015, partial η^2 ^= .070. Mean Visit Count was greater for the short-term (*M* = 16.568, *SE* = 0.479) than for the long-term (*M* = 14.900, *SE* = 0.473) condition. There was a significant interaction between Face Area and Location, *F*(4, 324) = 53.325, *p* < .001, partial η^2 ^= .397, as well as a significant but weak interaction between Face Area and Condition, *F*(4, 324) = 14.874, *p* < .001, partial η^2 ^= .155. Visit Count for the “cropped,” “border,” and “uncropped” areas was greater in the short-term condition than in the long-term condition (all *p*’s < .001), but it was greater for the “mouth” area in the long-term condition than in the short-term condition (*p* = .041). There was no significant interaction between Location and Condition, *F*(1, 81) = .090, *p* = .765, partial η^2 ^= .001. Face Area × Location × Condition interaction was significant but weak, *F*(4, 324) = 12.731, *p* < .001, partial η^2 ^= .136. In both conditions, there were no differences in mean Visit Count between the forehead versus chin areas for the “cropped” and the “mouth” ROIs. For the “border” and the “eyes” ROIs’, mean Visit Count was greater for the forehead- than for the chin-cropped faces in the short-term condition (*p*’s < .001), but not in the long-term condition. Visit Duration for the “noncropped” ROIs’ was greater for the chin- than for the forehead-cropped faces in both, the short-term (*p* < .001) and the long-term (*p* = .002), conditions. Figure 6 demonstrates the results of comparisons between mean Visit Count for different ROIs in forehead- and chin-cropped faces, separately for the two conditions.

#### Recognition analysis

**Figure 7.**
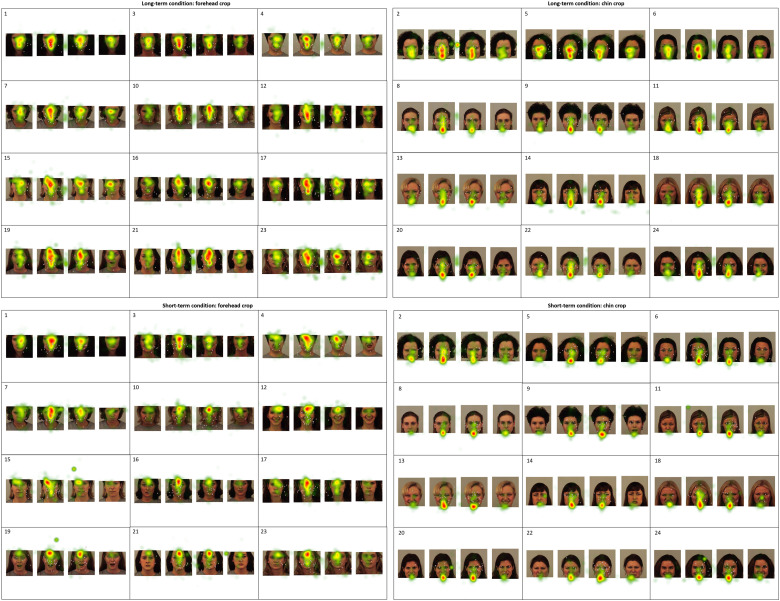
Recognition eye-tracking heatmaps representing relative Visit Duration and mouse clicks for the short-term and long-term conditions of the Faces Task.

Consistent with the behavioral data analysis, a mixed 2 × 2 × 2 ANOVA was conducted to assess the impact Error Type and Cropping Location on participants’ oculomotor variables across the two memory Conditions. The data were analyzed separately for Visit Duration and Visit Count. The adjustment for multiple comparisons was done with Bonferroni correction. The eye-tracking data were examined using the same ROIs as the behavioral Recognition analysis (Figure 2). The eye-tracking heatmap visualizations are presented in Figure 7.

**Figure 8.**
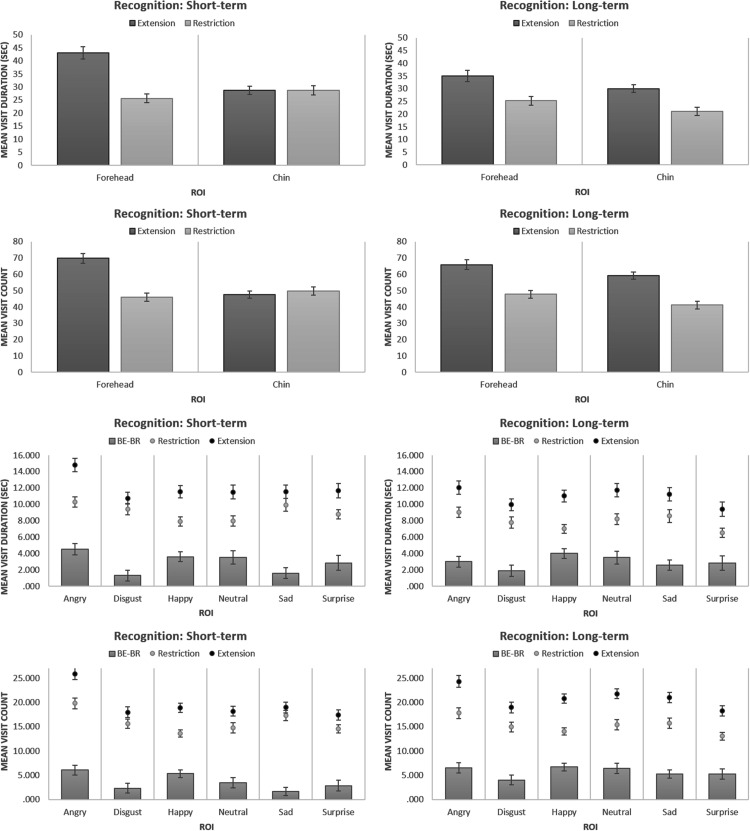
Recognition data: Visit Duration and Visit Count for the different types of errors in the Faces Task. ROI = Region of Interest; BE = Boundary Extension; BR = Boundary Restriction.

The analysis of *Visit Duration* revealed a substantial main effect for Error Type, *F*(1, 81) = 104.030, *p* < .001, partial η^2 ^= .562, demonstrating that the participants had higher mean Visit Duration for extension (*M* = 34.252, *SE* = 1.352) than for restriction (*M* = 25.161, *SE* = 1.026) answer options. There was a substantial main effect for cropping Location, *F*(1, 81) = 70.645, *p* < .001, partial η^2 ^= .466, showing that the participants had higher mean Visit Duration for the forehead-cropped (*M* = 32.265, *SE* = 1.259) than for the chin-cropped (*M* = 27.149, *SE* = 1.040) images. There was a significant Error Type × Location interaction, *F*(1, 81) = 31.698, *p* < .001, partial η^2 ^= .281. The difference between the mean Visit Duration for BE and BR answer options for the forehead-cropped images (*MD_BE-BR_* = 13.639, *SE* = 1.209, *p* < .001; *M*_*BE*_ = 39.085, *SE* = 1.654; *M*_*BR*_ = 25.446, *SE* = 1. 080) was greater than for the chin-cropped images (*MD_BE-BR_* = 4.543, *SE* = 1.197, *p* < .001; *M*_*BE*_ = 29.420, *SE* = 1.219; *M*_*BR*_ = 24.877, *SE* = 1.181). The effect of Condition was not significant, *F*(1, 81) = 2.791, *p* = .099, partial η^2 ^= .033. There was no Error Type × Condition interaction, *F*(1, 81) = .146, *p* = .703, partial η^2 ^= .002. There was no Location × Condition interaction, *F*(1, 81) = .677, *p* = .413, partial η^2 ^= .008. There was a significant Error Type × Location × Condition interaction between, *F*(1, 81) = 26.185, *p* < .001, partial η^2 ^= .244, indicating that the Error Type × Location interaction varied between the long-term and the short-term conditions. A separate analysis for the two conditions revealed that forehead versus chin asymmetry of BE effect (Error Type × Location interaction) was present in the short-term condition, *F*(1, 40) = 37.554, *p* < .001, partial η^2 ^= .484, but not in the long-term condition, *F*(1, 41) = .270, *p* = .606, partial η^2 ^= .007. Figure 8 presents recognition results for the different Error Types, Conditions and cropping Locations, based on eye-tracking data.

The analysis of *Visit Count* revealed a substantial main effect for Error Type, *F*(1, 81) = 131.129, *p* < .001, partial η^2 ^= .618, showing that the participants had higher mean Visit Count for the extension (*M* = 60.603, *SE* = 1.789) than for the restriction (*M* = 46.165, *SE* = 1.501) answer options. There was a substantial main effect for cropping Location, *F*(1, 81) = 85.516, *p* < .001, partial η^2 ^= .514, showing that the participants had higher mean Visit Count for the forehead-cropped (*M* = 57.355, *SE* = 1.671) than for the chin-cropped (*M* = 49.413, *SE* = 1.506) answer options. There was a significant Error Type × Location interaction, *F*(1, 81) = 39.501, *p* < .001, partial η^2 ^= .328. The difference between the mean Visit Count for BE and BR answer options for the forehead-cropped images (*MD*_*BE-BR*_ = 20.974, *SE* = 1.624, *p* < .001; *M*_BE_ = 67.842, *SE* = 2.144; *M*_BR_ = 46.868, *SE* = 1.518) was greater than for the chin-cropped images (*MD*_*BE-BR*_ = 7.903, *SE* = 1.645, *p* < .001; *M*_BE_ = 53.364, *SE* = 1.723; *M*_BR_ = 45.461, *SE* = 1.709). The effect of Condition was not significant, *F*(1, 81) = .006, *p* = .937, partial η^2 ^= .000. There was a significant but weak Error Type × Condition interaction, *F*(1, 81) = 8.249, *p* = .005, partial η^2 ^= .092. The Error Type effect was less pronounced in the short-term (*MD*_*BE-BR*_ = 10.817, *SE* = 1.794, *p* < .001; *M*_*BE*_ = 58.671, *SE* = 2.588; *M*_*BR*_ = 47.854, *SE* = 2.135) than in the long-term condition (*MD*_*BE-BR*_ = 18.060, *SE* = 1.772, *p* < .001; *M*_*BE*_ = 62.536, *SE* = 2.527; *M*_*BR*_ = 44.476, *SE* = 2.110). There was no interaction between Location and Condition, *F*(1, 81) = 2.085, *p* = .153, partial η^2 ^= .025. There was a significant Error Type × Location × Condition interaction, *F*(1, 81) = 38.500, *p* < .001, partial η^2 ^= .322. A separate analysis for the two conditions revealed that the forehead versus chin asymmetry of BE effect (Error Type × Location interaction) was present in the short-term condition, *F*(1, 40) = 54.347, *p* < .001, partial η^2 ^= .576, but not in the long-term condition, *F*(1, 41) = .005, *p* = .941, partial η^2 ^= .000. Figure 8 presents the recognition results for the different Error Types, Conditions and cropping Locations, based on the eye-tracking data.

#### The effect of Emotion

Consistent with the behavioral data analysis, a mixed 6 × 2 × 2 ANOVA was conducted to assess the impact of Emotion and Error Type on the eye-tracking measures, across the two memory Conditions. The data were analyzed separately for Visit Duration and Visit Count. The adjustment for the multiple comparisons was done with Bonferroni correction.

The analysis of *Visit Duration* revealed a substantial main effect of Error Type, *F*(1, 81) = 98.561, *p* < .001, partial η^2 ^= .549, demonstrating that the participants fixated at the BE answer options longer than at the BR answer options. It was a significant, though weak, effect of Emotion, *F*(5, 405) = 10.861, *p* < .001, partial η^2 ^= .118. Pairwise comparisons demonstrated that the faces representing “Angry” emotion evoked longer Visit Duration than all the other emotions (all *p*’s < .001, but for the “Sad” emotion *p* = .054). There was a significant but weak interaction between Emotion and Error Type, *F*(5, 405) = 4.216, *p* < .001, partial η^2 ^= .049, suggesting that the Error Type effect differed depending on the Emotion. Pairwise comparisons of the Emotion and Error Type interaction revealed that Mean Difference (BE-BR) was significant for all emotions: “Angry” (*MD_BE-BR_* = 3.771, *SE* = 0.470, *p* < .001); “Disgust” (*MD_BE-BR_* = 1.612, *SE* = 0.477, *p* = .001); “Happy” (*MD_BE-BR_* = 3.817, *SE* = 0.427, *p* < .001); “Neutral” (*MD_BE-BR_* = 3.524, *SE* = 0.564, *p* < .001); “Sad” (*MD_BE-BR_* = 2.113, *SE* = 0.451, *p* < .001); and “Surprise” (*MD_BE-BR_* = 2.869, *SE* = 0.622, *p* < .001) emotions. Furthermore, to compare the size of BE effect for different types of emotions, the additional repeated measures analysis with BE-BR as a dependent measure and Emotion as a within-subjects variable. Pairwise comparisons demonstrated that mean BE-BR for the “Angry” emotion was significantly greater compared with the “Disgust” (*MD* = 2.147, *SE* = 0.626, *p* = .014). BE-BR for the “Happy” emotion was also greater compared with the “Disgust” (*MD* = 2.204, *SE* = 0.601, *p* = .007) and “Sad” (*MD* = 1.700, *SE* = 0.554, *p* = .044) emotions. No other significant differences were revealed. The effect of Condition was not significant, *F*(1, 81) = 2.428, *p* = .123, partial η^2 ^= .029. There was no interaction between Type and Condition, *F*(1, 81) = .013, *p* = .909, partial η^2 ^< .001. There was a weak but significant interaction between Emotion and Condition, *F*(5, 81) = 2.780, *p* = .017, partial η^2 ^= .033, suggesting that the Emotion effect differed depending on the Condition. Figure 8 presents mean Visit Duration of BE and BR answers for the different types of Emotions in both Conditions. In addition, for a clearer demonstration of Error Type effect for each emotion, this figure displays the difference between BE and BR measures (BE-BR). The interaction between Emotion, Error Type, and Condition, *F*(5, 81) = 26.185, *p* < .001, partial η^2 ^= .244 was not significant.

Similarly, the analysis of *Visit Count* revealed a substantial main effect for Error Type, *F*(1, 81) = 121.330, *p* < .001, partial η^2 ^= .600, demonstrating that the participants fixated at the BE answer options more often than at the BR answer options. There was a significant effect for Emotion, *F*(5, 405) = 27.227, *p* < .001, partial η^2 ^= .252. Pairwise comparisons demonstrated that the faces representing “Angry” emotion evoked longer Visit Count than all the other emotions (all *MD_BE-BR_* > 3.705, *SE* < 0.689, *p*’s < .001). Visit Count for faces with the “Surprise” emotion was lower than the “Neutral” (*MD_BE-BR_* = − 1.684, *SE* = 0.527, *p* = .030) and “Sad” (*MD_BE-BR_* = −2.412, *SE* = 0.592, *p* = .002) emotions. There was a significant but weak interaction between Emotion and Error Type, *F*(5, 405) = 4.827, *p* < .001, partial η^2 ^= .056. The effect of Condition was not significant, *F*(1, 81) = .058, *p* = .811, partial η^2 ^= .001. There was a weak but significant interaction between Type and Condition, *F*(1, 81) = 6.102, *p* = .016, partial η^2 ^= .070. There was a weak but significant interaction between Emotion and Condition, *F*(5, 405) = 74.313, *p* = .024, partial η^2 ^= .031. Pairwise comparisons of the Emotion and Error Type interaction revealed that Mean Difference (BE-BR) was significant for all emotions: “Angry” (*MD_BE-BR_* = 6.274, *SE* = 0.732, *p* < .001); “Disgust” (*MD_BE-BR_* = 3.183, *SE* = 0.709, *p* < .001); “Happy” (*MD_BE-BR_* = 6.028, *SE* = 0.560, *p* < .001); “Neutral” (*MD_BE-BR_* = 4.934, *SE* = 0.751, *p* < .001); “Sad” (*MD_BE-BR_* = 3.472, *SE* = 0.582, *p* < .001); and “Surprise” (*MD_BE-BR_* = 3.997, *SE* = 0.769, *p* < .001) emotions. Furthermore, to compare the size of BE effect for different types of emotions, the additional repeated measures analysis with BE-BR as a dependent measure and Emotion as a within-subjects variable. Pairwise comparisons demonstrated that BE-BR for the “Angry” emotion was significantly greater compared with the “Disgust” (*MD* = 3.084, *SE* = 0.927, *p* = .020) and the “Sad” (*MD* = 2.783, *SE* = 0.880, *p* = .033) emotions. BE-BR for the “Happy” emotion was also greater compared with the “Disgust” (*MD* = 2.843, *SE* = 0.832, *p* = .015) and the “Sad” (*MD* = 2.542, *SE* = 0.764, *p* = .020) emotions. The interaction between Emotion, Error Type, and Condition, *F*(5, 405) = .908, *p* = .476, partial η^2 ^= .011, was not significant.

### Discussion

The analyses of behavioral (Error Frequency) and eye-tracking (Visit Duration and Visit Counts) Recognition data yielded highly consistent results. The main effect of Error Type was found: The BE answer options were selected significantly more frequently and attracted more attention than the BR answer options. Thus, Study 1 demonstrated the evidence of BE effect in face processing. The analysis revealed no interaction between Error Type and Condition for Error Frequency and Visit Duration measures; however, there was a weak but significant interaction for Visit Count measure. These results provide the robust evidence of the BE effect for the different retention intervals, though, Visit Count results indicate that this effect may be somewhat more conspicuous in the long-term condition*.* Furthermore, the BE effect was more pronounced for the forehead-cropped images. This demonstrates the evidence of the asymmetry of BE effect in face images. However, the forehead-biased asymmetry of BE effect manifested only in the short-term Condition. Markedly, during the Encoding, the forehead-biased asymmetry in Face Area effect was also found in the short-term, but not in the long-term Condition.

The additional analyses revealed the effect of Emotion on Visit Duration and Count: The “angry” emotion evoked more attention than all the other emotions. Furthermore, the found interaction between Emotion and Error type suggests that the strength of BE effect may differ depending on the type of Emotion. The analysis of the eye-tracking measures revealed that the BE effect was greater for the “Angry” and the “Happy” emotions and smaller for the “Disgust” and the “Sad” emotions. Similarly, but not fully consistent, the analysis of mouse clicks revealed that the BE effect was greater for the “Happy” emotion but smaller for the “Disgust” and the “Neutral” emotions.

## Study 2

After Study 1 has established BE in processing of cropped face images, Study 2 aimed to examine the relationship between the strength of BE and individual differences in imagery and emotion. Thus, the same participants as in the Study 1 were asked to perform additional tests on individual differences in object and spatial imagery and emotional processing.

### Method

#### Participants

Thirty-nine participants from the short-term memory condition and 38 participants from the long-term memory condition completed the full set of assessments.

#### Materials and procedure

The participants received four different questionnaires and tasks: OSIQ, Emotion Vividness task, Geneva Emotion Recognition Test (GERT), and Range and Differentiation of Emotional Experience Scale (RDEES). In addition, Emotion Recognition performance data from Faces Task (Study 1) were analyzed.

##### OSIQ

This is a self-report measure assessing individual differences in visual-object and visual-spatial imagery (Blajenkova et al., 2006). The OSIQ consists of 15 statements assessing object mental visualization and 15 statements assessing spatial mental visualization. Participants had to rate these items on a 5-point scale from total agreement to total disagreement. The scores for object and spatial imagery are calculated by averaging the corresponding 15 ratings per subscale. The internal reliabilities (Cronbach’s alpha) of the object and spatial imagery subscales are .83 and .79, respectively (Blajenkova et al., 2006).

##### Emotion Vividness task

In this task, participants had to imagine six basic emotions (anger, surprise, happiness, disgust, sadness, fear; see Ekman, 1992) as well as neutral facial expression inside the empty face outline, presented for 5 s on a computer screen. After imagining each emotion, participants rated the vividness of their subjective mental image using the 5-point scale adopted from the VVIQ (Marks, 1973): 5 perfectly clear and as vivid as normal vision), 4 (clear and reasonably vivid), 3 (moderately clear and vivid), 2 (vague and dim), 1 (no image at all, you only “know” that you are thinking of the object). This task was developed by the author of this study, and it is not a validated task of imagined emotion vividness.

##### GERT

This test is assessing the ability to accurately recognize emotional states (Schlegel, Grandjean, & Scherer, 2014). Participants watched 83 short video clips with sound, in which 5 male and 5 female actors expressed different emotions conveyed both by facial expressions and voice (using pseudolinguistic sentences). Participants had to select the emotion word (from 14 emotions), which best describes the emotion expresses in the video.

##### RDEES

This is self-report assessing individual differences in emotional complexity, which is defined as having emotional experiences that are broad in range and well-differentiated (Kang & Shaver, 2004). Seven items tap Range (e.g., “I have experienced a wide range of emotions throughout my life”), and seven Differentiation of Emotional Experiences (e.g., “I am aware of the subtle differences in the feelings that I have”). Participants rated each item on a 5-point scale (1 = does not describe me very well and 5 = describes me very well). The scores for Range (RDEES-r) and Differentiation (RDEES-d) subscales were computed by averaging the corresponding ratings. The internal reliability (Cronbach’s alpha) of this questionnaire is .85 (.82 for the RDEES-r subscale and .79 for RDEES-d subscale; Kang & Shaver, 2004).

##### Emotion Recognition

Based on the responses given in a Faces Task (Study 1), the accuracy of emotional recognition was computed as a number of correctly identified emotions. Note, this is not a validated task of emotional recognition, and also it was done simultaneously with memorization. The reliability was quite low (Cronbach’s alpha = .210). Thus, another variable, *Emotion Recognition*^Δ^ accuracy was computed by excluding the most inconsistent 4 items with accuracy below 50% (Cronbach’s alpha was still quite low = .259).

### Results

The relationships between different memory errors, imagery, and emotional measures were analyzed using Pearson’s Correlational Analysis. In the *short-term memory condition* (Table 1), the analysis revealed no relationship between the frequency of BE/BR errors and any of imagery measures. Emotion Recognition^Δ^ accuracy in the Faces Task tended to be positively correlated with BE and negatively with BR errors (*p*’s = .091). Furthermore, longer Visit Duration and Count (both for BE/BR images) were positively associated with higher scores on GERT (all *p*’s ≤ .015). Visit Count was negatively associated with Vividness of Emotional Imagery (*p* = .054 for Extension and *p* = .015 for Restriction Visit Count).

**Table 1.** Correlations Between all the Measures in the Short-Term Condition of the Faces Task.

	1	2	3	4	5	6	7	8	9	10	11	12	13
1. Extension errors	1.00												
2. Restriction errors	−1.00*	1.00											
3. Extension duration	.303†	−.303†	1.00										
4. Restriction duration	−.050	.050	.874*	1.00									
5. Extension count	.314†	−.314†	.873*	.793*	1.00								
6. Restriction count	−.056	.056	.717*	.853*	.876*	1.00							
7. OSIQ-object	−.011	.011	.050	.093	−.034	.015	1.00						
8. OSIQ-spatial	.048	−.048	.108	.066	−.157	−.212	.028	1.00					
9. Emotion vividness	.049	−.049	−.075	−.192	−.310†	−.386*	.232	.164	1.00				
10. GERT	.073	−.073	.420*	.437*	.461*	.495*	.241	−.281†	−.077	1.00			
11. RDEES-r	−.088	.088	.163	.232	.089	.153	.426*	−.077	.339*	.485*	1.00		
12. RDEES-d	.124	−.124	.008	.035	.058	.081	.401*	−.214	.295†	.148	.492*	1.00	
13. Emotion recognition	.141	−141	−.251	−.284†	−.150	−.193	−.071	−.161	.008	.016	−.029	.029	1.00
Emotion recognitionΔ	.275†	−.275†	−.016	−.066	.037	−.079	−.057	−.054	.049	.161	.086	.058	.870*

*Note*. OSIQ = Object-Spatial Imagery Questionnaire; GERT = Geneva Emotion Recognition Test; RDEES = Range and Differentiation of Emotional Experience Scale.

* *p* < .05. †*p* < .10.

In the *long-term memory condition* (Table 2), the analysis revealed significant positive relationship between the frequency of BE errors and object imagery (*p* = .038). Inversely, the relationship between the frequency of BR errors and object imagery was negative (*p* = .020). In addition, attention to BR images was significantly and negatively associated with object imagery (*p* = .039 for Visit Duration and *p* = .014 for Visit Count). Furthermore, RDEES-d was positively associated with frequency of BE Errors (*p* = .042), and negatively with frequency of BR Errors (*p* = .036), as well as restriction Visit Count (*p* = .025). The similar, but nonsignificant, trends were observed between the BE Errors and Emotion Vividness as well as GERT. Emotion Recognition accuracy in the Faces Task tended to be negatively associated with visual attention, both for BE/BR images (*p*’s ≤ .033 for Emotion Recognition^Δ^ and Visit Count, *p* = .032 for Visit Duration).

**Table 2.** Correlations Between all the Measures in the Long-Term Condition of the Faces Task.

	1	2	3	4	5	6	7	8	9	10	11	12	13
1. Extension errors	1.00												
2. Restriction errors	−1.000*	1.00											
3. Extension duration	.341*	−.341*	1.00										
4. Restriction duration	−.333*	.333*	.671*	1.00									
5. Extension count	.316†	−.316†	.826*	.519*	1.00								
6. Restriction count	−.398*	.398*	.512*	.868*	.580*	1.00							
7. OSIQ-object	.372*	−.372*	−.128	−.334*	−.146	−.406*	1.00						
8. OSIQ-spatial	−.032	.032	.174	−.019	.077	−.017	−.231	1.00					
9. Emotion vividness	.267	−.267	−.033	−.140	−.093	−.143	.322*	.035	1.00				
10. GERT	.186	−.186	.082	.003	.147	.115	.117	−.075	.253	1.00			
11. RDEES-r	.228	−.228	−.054	−.119	−.124	−.235	.451*	−.068	.095	.036	1.00		
12. RDEES-d	.335*	−.335*	−.115	−.254	−.223	−.425*	.562*	−.096	.135	−.056	.677*	1.00	
13. Emotion recognition	.104	−.104	−.114	−.202	−.195	−.344*	.439*	.005	−.003	.083	.010	.145	1.00
Emotion recognitionΔ	.119	−.119	−.184	−.315†	−.323*	−.462*	.378*	−.059	−.052	.079	−.141	.142	.856*

† *p* < .10. *p < .05.

*Note*. OSIQ = Object-Spatial Imagery Questionnaire; GERT = Geneva Emotion Recognition Test; RDEES = Range and Differentiation of Emotional Experience Scale.

In addition, the relationship between imagery and emotional measures was examined using the combined data from the long-term and short-term conditions (*N* = 79). Object imagery scale of the OSIQ was positively correlated with Emotion Vividness (*r* = .289, *p* = .010), RDEES-r (*r* = .439, *p* < .001), RDEES-d (*r* = .508, *p* < .001), as well as with Emotion Recognition accuracy in the Faces Task (*r* = .223, *p* = .049)/Emotion Recognition^Δ^ (*r* = .193, *p* = .091). GERT was positively correlated with RDEES-r (*r* = .268, *p* = .018), but negatively and marginally significantly with spatial imagery scale of the OSIQ (*r* = −.215, *p* = .058). Emotion Vividness tended to correlate with RDEES-r (*r* = .216, *p* = .058) and RDEES-d (*r* = .220, *p* = .053), and the latter two were also interrelated (*r* = .589, *p* < .001).

### Discussion

Study 2 demonstrated positive relationship between BE measures and individual differences in object but not spatial imagery. This indicates that individuals with higher object imagery showed greater BE and lesser BR effect. Similarly, individuals who reported higher emotional differentiation tended to have more pronounced BE errors. However, the relation between BE and imagery or emotional measures was observed primarily in the long-term condition. Consistent with the previous research (Blazhenkova & Kozhevnikov, 2010), object, but not spatial imagery, tended to be positively associated with different emotional measures, which tended to be interrelated. Overall, object imagery and emotional measures’ relationships with BE measures were similar.

## General Discussion

The present work examined BE phenomenon in face processing. The results of the Study 1 demonstrated significantly more BE than restriction recognition errors, as revealed by both performance and eye-tracking measures. Thus, consistent with previous literature (Hubbard et al., 2010; Intraub & Bodamer, 1993), the present findings suggest that people are prompted to perceptual and memory errors, in which they see or remember seeing parts of images that were not visible during the presentation but were possible to be inferred from the visible information. The present study *demonstrated BE effect in both short-term and long-term memory conditions*. This is consistent with other research that found BE for a wide range of retention intervals (Intraub & Dickinson, 2008; Intraub & Richardson, 1989; Safer et al., 1998).

The present results indicate that BE effect could be somewhat *more pronounced in the long-term condition*. The found difference in BE effect between the two memory conditions was rather small and appeared only in Visit Count analysis. From the multisource model perspective (Intraub, 2012), this finding may suggest that the increased retention time could lead to a greater difficulty in distinguishing between amodally generated and remembered visual sensory information, thus resulting in a greater BE in the long-term condition. In addition, the difference between the two memory conditions may be explained by the difference in the awareness about the nature of the memory task. In the short-term condition, after the very first trial, participants became aware of the subsequent memory test that required boundary knowledge, whereas in the long-term condition, participants became aware about the memory test only after the first block of trials. Moreover, due to a significant delay between the Encoding and Recognition in the long-term condition, the awareness about memorization demand during the Encoding could decrease. It is important to note that during the Encoding, participants had to simultaneously identify the emotion and to memorize the face, which could be considered as a dual task (Pashler, 1994). Emotional identification was a primary task, and Recognition came afterward. Eye-tracking data indicated that the participants, first, focused on the emotional identification. The majority of the first fixations were in the “eyes” area, whereas other facial parts were examined later. Because the differences between the Recognition answer options appeared solely in the place of a crop, the “border” area provided important cues for face memorization, critical for the subsequent Recognition. As it can be seen from the eye-tracking visualizations, with more trials, participants increased their attention toward the “border” areas, most probably because they were realizing the importance of this area for the Recognition performance. Indeed, several participants spontaneously mentioned that they were focused on border areas in demand to the expected subsequent Recognition task. Similarly, Gagnier et al. (2013) suggested that the cropped region is a salient marker of boundary placement and found multiple fixations to this area. In the long-term condition, the Recognition was delayed, so, possibly, the participants were more focused on emotional identification and less focused on the memorization for further Recognition task. Indeed, in the short-term condition, the attention to the “border” and its neighboring “cropped” areas was greater than in the long-term condition, while the attention to the “eyes” and “mouth” areas was greater in the long-term condition. These results are similar to Gagnier et al. (2013) finding that the participants who were told that the boundary memory was tested had higher oculomotor activity near the boundaries of pictures than the participants who were not informed about the nature of the test. Thus, consistent with previous research that showed the reduction of BE error when participants were informed about the nature of the task (Gagnier et al., 2013; Intraub & Bodamer, 1993), the short-term memory condition in the present research yielded somewhat reduced BE error.

The present research *demonstrated BE for isolated face images on blank backgrounds*. This is unlike the majority of the studies, which found BE primarily for images containing scenes with background texture but not in response to isolated objects removed from the background context (Chadwick et al., 2013; Gottesman & Intraub, 2002, 2003; Intraub et al., 1998; Mullally et al., 2012). It should be noted that stimuli used in the present work contained cropped faces, which are likely to elicit the sense of continuation beyond the picture boundaries. Thus, the perception of close-up cropped faces yields BE, as the perception of scenes does. Probably, cropped faces, similar to truncated scenes, elicit BE, which may be not true for other types of nonface objects. Further research is needed to examine whether this effect could be found for different nonface objects. The present findings advocate some common mechanisms in face and scene perception. As suggested by previous literature, the perception of faces involves configural processing, for example, a perception of the relations between the separate features rather than their processing in piecemeal or analytical way (Tanaka & Farah, 1993; Tanaka & Sengco, 1997). Configural processing of faces implies holistic processing (building a gestalt from separate individual features), as well as relational arrangement of features (two eyes above the nose and a mouth below) and distances between these features (Maurer, Grand, & Mondloch, 2002). Similarly, scene perception involves configural and relational processing (Chun, 2003). Furthermore, Gallagher et al. (2005) found that the salience of the scene background increases the magnitude of BE. Indeed, a large body of evidence advocates that faces are extremely salient visual stimuli (Farah et al., 1998). Besides, it may be the case that face images elicited BE in the current study because the stimuli represented close-up views. As indicated by previous research (Intraub et al., 1992), the degree of BE grows as increasingly close-up views are presented.

Furthermore, the present findings provide a new *evidence on asymmetry of BE*. Consistent with predictions, the results of the Study 1 demonstrated significantly more pronounced BE errors for forehead- than for chin-cropped face area. The Recognition analysis showed that, overall, the measures of BE in the forehead-cropped faces exceeded the measures of BE in the chin-cropped faces. In support of the attentional explanation of the asymmetry in BE, the present work found the asymmetry in attention in processing different facial parts. The present findings are consistent with the literature on face processing, showing that greater attention is paid to the upper part of the face, and in particular, to eyes area (Janik et al., 1978; McKelvie, 1976; Mertens et al., 1993; Pellicano et al., 2006; Walker-Smith et al., 1977; Yarbus, 1967). The eye-tracking analysis of the Encoding demonstrated that, in both memory conditions, for both forehead- and chin-cropped faces, most attention was paid to the “eyes” and then “mouth” regions. Possibly, the presence of eyes may cue the attention to the neighboring “border” areas. The attentional cueing by the “eyes” may elicit BE in the upper part of the face and suppress it on the uncued side (see also Intraub et al., 2006). The present findings that BE was greater for the upper than the lower part the face may be explained by the increased attention to the “eyes” area. Surprisingly, while the “eyes” was the most salient area in both conditions, the asymmetry of BE effect was evident only in the short-term, but not in the long-term condition. It is possible that with a more delayed recognition testing, the memory representation may include less visual information and more information suggested by the mental schema. The enhanced visual attention in the upper part of a face may trigger the forehead-biased asymmetry of BE. However, visual detail may be not remembered after a significant delay, thus the asymmetry of BE could be observed only in the short-term, but not in the long-term condition. Also, the intriguing difference in asymmetry of BE between the two memory conditions could be related to the difference in attentional distribution during the Encoding. The analysis of oculomotor behavior during the Encoding demonstrated that in the short-term condition, the “eyes” areas in the forehead-cropped faces attracted more attention than in the chin-cropped faces. Even when excluding “eyes,” the upper “border” areas attracted more attention than the lower “border” face areas. Besides, “noncropped” upper face areas attracted more attention than “noncropped” lower face areas. Markedly, this increased attention to the upper face parts in the forehead-cropped compared with chin-cropped faces was found only in the short-term condition. This indicates that the differences in processing forehead- and chin-cropped faces between the two memory conditions already appeared during the Encoding. Overall, the earlier comparisons between the two memory conditions suggest that the specific patterns of BE errors, that is, forehead-biased asymmetry, revealed in memory (during the Recognition) were consistent with the specific patterns of attention revealed during the perception (Encoding).

Besides the attentional asymmetry in face processing, there are other possible explanations why such an asymmetry in BE in face processing may exist. One possibility could be that in our everyday life, we are more used to see forehead- than chin-cropped faces (e.g., people wearing hats); due to this experience, we may be better in mental continuation of the upper parts rather than lower parts of faces. This possibility could be explored by examining BE in face images of human-like animals (e.g., monkeys), who do not typically cover their heads. Furthermore, because previous research found that asymmetry in scene representation may be caused by the anticipatory processing in a certain direction, and in particular, anticipated motion (Courtney & Hubbard, 2004), it is possible that “eyes” may convey some sort of motion information, causing BE. There is evidence that gaze direction may indicate anticipated action and intention (Klin, Jones, Schultz, & Volkmar, 2003). Additional research is needed to explore the possibility whether gaze direction (e.g., looking to the left or to the right side, looking up or down, closed or opened eyes) may influence the asymmetry of BE. Alternatively, a greater BE of the upper face part could be because the upper face area is greater in size than the lower one. Experiments, using artificially modified faces with equalized forehead and chin area, can address this possibility. Another possibility, which also can be tested by using artificially modified face stimuli, is that the asymmetry of BE in faces may be caused by the perceptual differences between the top of the head, which does not continue, and the chin area, which extends to the neck and below body parts. Further research is necessary to determine which explanation is best supported. Notably, because both conditions used the same face stimuli, the found difference in the asymmetry of BE between the two conditions indicates that this asymmetry could not fully be due to a bias caused by the characteristics of the stimuli. Irrespective of why such an asymmetry may occur, the present finding of asymmetry of BE in face images expands the existing evidence that the content of an image may influence BE. Furthermore, it contributes to understanding the roles of perception and memory in BE phenomenon.

The results of Study 2 showed that *individual differences in imagery relate to BE*. In particular, object, but not spatial, imagery was associated with BE. These findings support our hypothesis that, depending on the content of the image (scene vs. isolated object or face), BE may relate not only to the strength of *spatial* imagery, as in Munger and Multhaup (2016) study, which used scenes, but also to the strength of *object* imagery, as in the present study, which used faces. Besides, the present results demonstrated that object imagery was positively associated with vividness of imagined emotional expressions, which requires visualizing pictorial perceptual details inside the empty face contour.

Emotion vividness, similar to object imagery, tended to be positively associated with extension and negatively with restriction errors. The present results extend the research of Mullally et al. (2012), who found that individual differences in spatial scene imagery vividness was associated with BE for scenes, whereas the present findings show the association between object imagery and BE for faces. In addition to measures of individual differences in imagery, the current research included measures of emotional processing. Consistent with expectations and previous research (Blazhenkova & Kozhevnikov, 2010), emotional measures were positively related with object but not with spatial imagery. Similar to object imagery, the emotional measures tended to have positive relationship with BE errors. Intriguingly, the relationships between the BE measures and individual differences in imagery were evident mostly in the long-term condition. Thus, the individuals with greater imagery abilities tended to have a greater BE. Probably, because imagery involves a top-down mental constructive processing (Ganis et al., 2004; Mechelli et al., 2004), these individuals were more likely to make commission memory errors. The relationship between BE and imagery could manifest only in the long-term condition because a delayed recognition testing task required less sensory information and more mental schema suggestions. Previous explanations of BE mechanisms indicated the involvement of mental imagery or mental visualization of nonvisible image parts, extending beyond the borders. Foley, Foley, Scheye, and Bonacci (2007) indicated the resemblance of BE errors to object completion errors, resulting from mental filling-in. Perceptual filling-in involves mental completion of visual image and the perception of visual features that are physically present only the surroundings (Komatsu, 2006; Walls, 1954). It is phenomenologically experienced as a perception of the completed textured and colorful objects (Kanizsa, 1979; Ramachandran & Gregory, 1991) and having a coherent object representation (Shipley & Kellman, 1992; Yantis, 1995). However, previous research that showed that BE for scenes occurred regardless of whether a boundary cropped an object or not, ruled out the object completion explanation of BE (Intraub et al., 1992; Intraub & Bodamer, 1993; Intraub & Richardson, 1989). Besides, Intraub et al. (1998) indicated that imagination of background outward of central objects (visual-spatial information) resulted in BE, whereas imagination of the color within the central object (visual-object information) did not. In contrast to scene images, possibly, in case of face cropped images, the anticipatory representation of the cropped face part may cause the expectation of a continuation of the face itself (a central cropped object), rather than a scene background outward the face, thus it may involve mental completion processes. Research showed that eye scanpaths during visual imagery may reenact those of perception and reflect visual scene content (Brandt, & Stark, 1997; Laeng, & Teodorescu, 2002; Mast, & Kosslyn, 2002). Thus, it is probable that the eye fixations in the cropped areas indicate that participants mentally visualized the missing facial part. As can be seen from the eye-tracking encoding heat maps (Study 1), in the cropped areas, participants were mostly focused inside the anticipated missing face part rather in the background of a scene outward the face. As suggested by Gottesman and Intraub (2003), image boundaries can be considered a partial occluder of a larger scene that extends behind it. In case of amodal completion, the information about the occluded parts of an object is not supported by sensory modalities. Hubbard et al. (2010) proposed that, although object completion is unlikely to be the cause of BE, BE might induce amodal continuation/completion of a cropped object, as in case of a partially occluded object (Hubbard et al., 2010).

Gottesman and Intraub (2002) proposed that amodal continuation of surfaces plays fundamental role in scene perception, especially for surfaces truncated by boundaries. They claimed that spatial extrapolation does not require detailed and rich background but is just the suggestion of a continuity beyond the image boundaries. However, it is possible that in case of close-up face images, BE may involve some sort of modal completion rather than (or in addition to) amodal continuation. Nonspatial object imagery, which refers to visualization of sensory surface properties such as color and texture, may be more involved in extrapolation of a cropped *face* image beyond the boundaries. Future efforts to establish the nature of mental extrapolation in processing of cropped face images could provide insight about the characteristics of imagery involved in BE. The current study brings a question whether the scene construction schema activated by a limited view is purely spatial and how it depends on the content of the scene. While the existing neuroimaging research indicates that BE is supported by the scene-selective brain regions but not by those specific to object recognition (Park et al., 2007), the present results raise a question whether BE of cropped face images may be underpinned by brain areas involved in visual-object processing. Further research is needed to examine the cognitive mechanisms and neural underpinnings of BE during cropped face processing. The current literature indicates the existence of both visual-spatial and visual-object aspects in scene representations. A dual-path model of scene processing, based on the neurological “what” versus “where” distinction (Ungerleider & Haxby, 1994), dissociate between processing object and spatial information (Chun, 2003). Similarly, Aguirre and D’Esposito (1997) distinguished between processing of large-scale environmental scene pictorial appearance (e.g., landmark knowledge) and spatial locations within a scene (e.g., survey knowledge), subserved by distinct ventral “what” and dorsal “where” neural pathway. Although visual-spatial properties such as position were shown to be better encoded in scene representations than visual-object surface properties such as color (Aginsky & Tarr, 2000), object visualization may play a significant role in scene processing and BE, depending on scene content. The present work adds a new dimension to the research on BE and encourages the future investigation on individual differences in object versus spatial imagery and emotion in relation to BE depending on stimuli type.

Furthermore, the exploratory analyses of the effect of different emotions on BE revealed mixed findings. Faces with “Angry” and “Happy” emotions tended to evoke a greater boundary extrapolation. These results are inconsistent with previous research that showed no difference in BE for images with different emotional content (Candel et al., 2003, 2004). They are only partially consistent with the research that demonstrated BE only for scenes with positive but not negative emotional content (Ménétrier et al., 2013; Safer et al. 1998). Such a discrepancy in findings could be due to the nature of the stimuli that may mobilize different cognitive mechanisms. In particular, Candel et al. as well as Safer et al. used neutral and aversive scenes that evoked highly unpleasant and arousing emotions, whereas the present study used faces and the task that involved emotional recognition. Ménétrier et al. used dynamic stimuli representing human bodies cropped in the midthigh or knee level, whereas the present study used static pictures representing cropped faces. The present findings suggest that both positive and negative emotions may induce BE. Notably, both Angry and Happy emotions are categorized as “aroused,” while disgust and sad are “not aroused” (Russel, 1980). Possibly, the level or arousal may affect the strength of BE. Contrary to the suggestion that BE may be “immune to emotional content because it involves early perceptual processes, rather than relatively late reconstructive memory processes” (Candel et al., 2003, pp. 694–695), short-term and long-term memory conditions produced similar effects of emotion on BE. However, present results should be treated with caution due to a small number of stimuli per Emotion. Future studies are needed to examine the effect of different emotions on spatial extrapolation in face stimuli.

One of the limitations of the present study was using only female faces as stimuli. Unlikely, but possibly, the found results may not be observed for stimuli using faces of males. The investigation of this possibility requires an additional investigation. For the present data, the comparison between males and females on BE and BR measures (mean Error Frequencies, Visit Duration, and Count) did not reveal any significant sex differences. Another limitation was that presentation of Recognition answer options was not exactly the same for the short-term (half increased from right to left, half increased from left to right) and the long-term condition (all increased from right to left). This discrepancy was due to the fact that the long-term condition was developed first. After the consideration of the long-term memory limitations, the research design was improved in the short-term condition. Despite this difference, the results indicate the evidence of BE for both orders of the Recognition choices. Furthermore, the present study did not use any deliberate imagery instructions, and during the encoding, participants were prompted to focus on recognition of facial expressions and face memorization. As suggested by previous research, spontaneous imagery, in contrast to deliberately induced imagery, may encourage source memory errors (Foley et al., 2010). Possibly, such an instruction amplified the found BE effect. Further research is needed to examine the effects of imagery instructions on BE, manipulating not only deliberate versus spontaneous imagery but also object versus spatial imagery instructions.

In sum, the current work presented new evidence on BE in face processing. Furthermore, it demonstrated that for short retention intervals, BE errors were more pronounced for forehead, than for chin face areas, which provided a new evidence of asymmetry in BE. Moreover, individual differences in emotional ability and object, but not spatial, imagery were found to be positively associated with BE in face processing. This investigation raises a number of research questions and highlights future research directions, which could elucidate the nature of BE phenomenon.

## Notes

1 Eighty-four participants took part in this research. However, one participant was excluded from the analysis due to a substantial data loss (responses for only 17 out of 24 trials were recorded).

2 A mouse click was not recorded for the Recognition trial #6 in the long-term condition. The missing data for this trial was recovered based on the eye-tracking data. Due to the high correspondence between the mouse click decisions and visit duration, the recognition choice response was assumed to correspond to the longest visit duration.

3 The analysis was not computed for the main effects of Location and Condition, as well as for Location × Condition interaction because in the given experimental design, the number of stimuli cropped in the forehead and chin was the same for the both conditions.

4 The analysis was not computed for the main effects of Emotion and Condition, as well as for Emotion × Condition interaction because in the given experimental design, the number of choices for each emotion was the same for the both conditions.

## References

[bibr1-2041669517724808] Aginsky V. Tarr M. J. (2000) How are different properties of a scene encoded in visual memory? Visual Cognition 7: 147–162.

[bibr2-2041669517724808] Aguirre G. K. D’Esposito M. (1997) Environmental knowledge is subserved by separable dorsal/ventral neural areas. The Journal of Neuroscience 17: 2512–2518. 9065511 10.1523/JNEUROSCI.17-07-02512.1997 PMC6573507

[bibr3-2041669517724808] Atkinson R. C. Shiffrin R. M. (1968) Human memory: A proposed system and its control processes. In: Spence R. W. Spence J. T. (eds) The psychology of learning and motivation Vol. 2, New York, NY: Academic, pp. 89–195.

[bibr4-2041669517724808] Baddeley A. D. Hitch G. (1974) Working memory. In: Bower G. H. (ed.) The psychology of learning and motivation Vol. 8, San Diego, CA: Academic Press, pp. 47–89.

[bibr5-2041669517724808] Baddeley A. D. Warrington E. K. (1970) Amnesia and the distinction between long-and short-term memory. Journal of Verbal Learning and Verbal Behavior 9: 176–189.

[bibr6-2041669517724808] Bird C. M. Burgess N. (2008) The hippocampus and memory: Insights from spatial processing. Nature Reviews Neuroscience 9: 182–194. 18270514 10.1038/nrn2335

[bibr7-2041669517724808] Blajenkova O. Kozhevnikov M. Motes M. A. (2006) Object-spatial imagery: A new self-report imagery questionnaire. Applied Cognitive Psychology 20: 239–263.

[bibr8-2041669517724808] Blazhenkova O. (2016) Vividness of object and spatial imagery. Perceptual and Motor Skills 122: 490–508. 27166329 10.1177/0031512516639431

[bibr9-2041669517724808] Blazhenkova O. Kozhevnikov M. (2010) Visual-object ability: A new dimension of non-verbal intelligence. Cognition 117: 276–301. 20887982 10.1016/j.cognition.2010.08.021

[bibr10-2041669517724808] Brandt S. A. Stark L. W. (1997) Spontaneous eye movements during visual imagery reflect the content of the visual scene. Journal of Cognitive Neuroscience 9: 27–38. 23968178 10.1162/jocn.1997.9.1.27

[bibr11-2041669517724808] Candel, I., Merckelbach, H., Houben, K., & Vandyck, I. (2004). How children remember neutral and emotional pictures: boundary extension in children s scene memories. *American Journal of Psychology*, *117*, 249–257. 15209372

[bibr12-2041669517724808] Candel I. Merckelbach H. Zandbergen M. (2003) Boundary distortions for neutral and emotional pictures. Psychonomic Bulletin & Review 10: 691–695. 14620365 10.3758/bf03196533

[bibr13-2041669517724808] Carlesimo G. A. Fadda L. Turriziani P. Tomaiuolo F. Caltagirone C. (2001) Selective sparing of face learning in a global amnesic patient. Journal of Neurology, Neurosurgery & Psychiatry 71: 340–346. 10.1136/jnnp.71.3.340 PMC1737550 11511707

[bibr14-2041669517724808] Chadwick M. J. Mullally S. L. Maguire E. A. (2013) The hippocampus extrapolates beyond the view in scenes: An fMRI study of boundary extension. Cortex 49: 2067–2079. 23276398 10.1016/j.cortex.2012.11.010 PMC3764338

[bibr15-2041669517724808] Chun M. M. (2003) Scene perception and memory. Psychology of Learning and Motivation 42: 79–108.

[bibr16-2041669517724808] Courtney, J. R., & Hubbard, T. L. (2004, November). *Possible asymmetries and effects of attention in boundary extension*. Paper presented at the 45th Annual Meeting of the Psychonomic Society, Minneapolis, MN.

[bibr17-2041669517724808] Daniels K. K. Intraub H. (2006) The shape of a view: Are rectilinear views necessary to elicit boundary extension? Visual Cognition 14: 129–149.

[bibr18-2041669517724808] Dickinson C. A. Intraub H. (2008) Transsaccadic representation of layout: What is the time course of boundary extension? Journal of Experimental Psychology: Human Perception and Performance 34: 543. 18505322 10.1037/0096-1523.34.3.543 PMC2754043

[bibr19-2041669517724808] Dickinson C. A. Intraub H. (2009) Spatial asymmetries in viewing and remembering scenes: Consequences of an attentional bias? Attention, Perception, & Psychophysics 71: 1251–1262. 10.3758/APP.71.6.1251 PMC2792631 19633341

[bibr20-2041669517724808] Dobson M. Markham R. (1993) Imagery ability and source monitoring: Implications for eyewitness memory. British Journal of Psychology 84: 111–118. 8467368 10.1111/j.2044-8295.1993.tb02466.x

[bibr21-2041669517724808] Ekman P. (1992) An argument for basic emotions. Cognition & Emotion 6: 169–200.

[bibr22-2041669517724808] Ellis H. D. (1975) Recognizing faces. British Journal of Psychology 66: 409–426. 1106805 10.1111/j.2044-8295.1975.tb01477.x

[bibr23-2041669517724808] Epstein R. Kanwisher N. (1998) A cortical representation of the local visual environment. Nature 392: 598–601. 9560155 10.1038/33402

[bibr24-2041669517724808] Farah M. J. (1988) Is visual imagery really visual? Overlooked evidence from neuropsychology. Psychological Review 95: 307. 3043530 10.1037/0033-295x.95.3.307

[bibr25-2041669517724808] Farah M. J. Hammond K. M. Levine D. N. Calvanio R. (1988) Visual and spatial mental imagery: Dissociable systems of representation. Cognitive Psychology 20: 439–462. 3191667 10.1016/0010-0285(88)90012-6

[bibr26-2041669517724808] Farah M. J. Wilson K. D. Drain M. Tanaka J. N. (1998) What is “special” about face perception? Psychological Review 105: 482. 9697428 10.1037/0033-295x.105.3.482

[bibr27-2041669517724808] Foley A. M. Foley H. J. Scheye R. Bonacci A. M. (2007) Remembering more than meets the eye: A study of memory confusions about incomplete visual information. Memory 15: 616–633. 17654277 10.1080/09658210701450919

[bibr28-2041669517724808] Foley M. A. Foy J. Schlemmer E. Belser-Ehrlich J. (2010) Imagery encoding and false recognition errors: Examining the role of imagery process and imagery content on source misattributions. Memory 18: 801–821. 20924947 10.1080/09658211.2010.509731

[bibr29-2041669517724808] Freyd J. J. (1983) The mental representation of movement when static stimuli are viewed. Perception & Psychophysics 33: 575–581. 6622194 10.3758/bf03202940

[bibr30-2041669517724808] Freyd J. J. Finke R. A. (1984) Representational momentum. Journal of Experimental Psychology: Learning, Memory, and Cognition 10: 126. 10.1037//0278-7393.14.1.112 2963890

[bibr31-2041669517724808] Futterweit L. R. Beilin H. (1994) Recognition memory for movement in photographs: A developmental study. Journal of Experimental Child Psychology 57: 163–179. 8169580 10.1006/jecp.1994.1008

[bibr32-2041669517724808] Gagnier K. M. Dickinson C. A. Intraub H. (2013) Fixating picture boundaries does not eliminate boundary extension: Implications for scene representation. The Quarterly Journal of Experimental Psychology 66: 2161–2186. 23547787 10.1080/17470218.2013.775595 PMC4551391

[bibr33-2041669517724808] Gallagher, K., Balas, B., Matheny, J., & Sinha, P. (2005) The effects of scene category and content on boundary extension. In B. Bara, L. Barsalou & M. Bucciarelli (Eds.), *Proceedings of the 27th Annual Meeting of the Cognitive Science Society*. Stresa, Italy: Cognitive Science Society.

[bibr34-2041669517724808] Ganis G. Thompson W. L. Kosslyn S. M. (2004) Brain areas underlying visual mental imagery and visual perception: An fMRI study. Cognitive Brain Research 20: 226–241. 15183394 10.1016/j.cogbrainres.2004.02.012

[bibr35-2041669517724808] Goldstein A. G. Mackenberg E. J. (1966) Recognition of human faces from isolated facial features: A developmental study. Psychonomic Science 6: 149–150.

[bibr36-2041669517724808] Gonsalves B. Paller K. A. (2000) Neural events that underlie remembering something that never happened. Nature Neuroscience 3: 1316–1321. 11100153 10.1038/81851

[bibr37-2041669517724808] Gonsalves B. Reber P. J. Gitelman D. R. Parrish T. B. Mesulam M. M. Paller K. A. (2004) Neural evidence that vivid imagining can lead to false remembering. Psychological Science 15: 655–660. 15447635 10.1111/j.0956-7976.2004.00736.x

[bibr38-2041669517724808] Gottesman C. V. Intraub H. (1999) Wide-angle memories of close-up scenes: A demonstration of boundary extension. Behavior Research Methods, Instruments, & Computers 31: 86–93. 10.3758/bf03207697 10495838

[bibr39-2041669517724808] Gottesman C. V. Intraub H. (2002) Surface construal and the mental representation of scenes. Journal of Experimental Psychology: Human Perception and Performance 28: 589. 12075890 10.1037//0096-1523.28.3.589

[bibr40-2041669517724808] Gottesman C. Intraub H. (2003) Constraints on spatial extrapolation in the mental representation of scenes: View-boundaries vs. object-boundaries. Visual Cognition 10: 875–893.

[bibr41-2041669517724808] Grill-Spector K. Kushnir T. Edelman S. Avidan G. Itzchak Y. Malach R. (1999) Differential processing of objects under various viewing conditions in the human lateral occipital complex. Neuron 24: 187–203. 10677037 10.1016/s0896-6273(00)80832-6

[bibr42-2041669517724808] Haxby J. V. Gobbini M. I. Furey M. L. Ishai A. Schouten J. L. Pietrini P. (2001) Distributed and overlapping representations of faces and objects in ventral temporal cortex. Science 293: 2425–2430. 11577229 10.1126/science.1063736

[bibr43-2041669517724808] Hubbard T. L. (1996) Displacement in depth: Representational momentum and boundary extension. Psychological Research 59: 33–47. 8693049 10.1007/BF00419832

[bibr44-2041669517724808] Hubbard T. L. Hutchison J. L. Courtney J. R. (2010) Boundary extension: Findings and theories. The Quarterly Journal of Experimental Psychology 63: 1467–1494. 20432178 10.1080/17470210903511236

[bibr45-2041669517724808] Hyman I. E. Jr. Pentland J. (1996) The role of mental imagery in the creation of false childhood memories. Journal of Memory and Language 35: 101–117.

[bibr46-2041669517724808] Intraub H. (1997) The representation of visual scenes. Trends in Cognitive Sciences 1: 217–222. 21223910 10.1016/S1364-6613(97)01067-X

[bibr47-2041669517724808] Intraub H. (2010) Rethinking scene perception: A multisource model. Psychology of Learning and Motivation 52: 231–264.

[bibr48-2041669517724808] Intraub H. (2012) Rethinking visual scene perception. Wiley Interdisciplinary Reviews: Cognitive Science 3: 117–127. 26302476 10.1002/wcs.149

[bibr49-2041669517724808] Intraub H. Bender R. S. Mangels J. A. (1992) Looking at pictures but remembering scenes. Journal of Experimental Psychology: Learning, Memory, and Cognition 18: 180. 10.1037//0278-7393.18.1.180 1532019

[bibr50-2041669517724808] Intraub H. Bodamer J. L. (1993) Boundary extension: Fundamental aspect of pictorial representation or encoding artifact? Journal of Experimental Psychology: Learning, Memory, and Cognition 19: 1387. 10.1037//0278-7393.19.6.1387 8270890

[bibr51-2041669517724808] Intraub H. Daniels K. K. Horowitz T. S. Wolfe J. M. (2008) Looking at scenes while searching for numbers: Dividing attention multiplies space. Perception & Psychophysics 70: 1337–1349. 18927017 10.3758/PP.70.7.1337 PMC4551389

[bibr52-2041669517724808] Intraub H. Dickinson C. A. (2008) False memory 1/20th of a second later what the early onset of boundary extension reveals about perception. Psychological Science 19: 1007–1014. 19000211 10.1111/j.1467-9280.2008.02192.x PMC2792630

[bibr53-2041669517724808] Intraub H. Gottesman C. V. Bills A. J. (1998) Effects of perceiving and imagining scenes on memory for pictures. Journal of Experimental Psychology: Learning, Memory, and Cognition 24: 186. 10.1037//0278-7393.24.1.186 9438959

[bibr54-2041669517724808] Intraub H. Gottesman C. V. Willey E. V. Zuk I. J. (1996) Boundary extension for briefly glimpsed photographs: Do common perceptual processes result in unexpected memory distortions? Journal of Memory and Language 35: 118–134.

[bibr55-2041669517724808] Intraub H. Hoffman J. E. Wetherhold C. J. Stoehs S. A. (2006) More than meets the eye: The effect of planned fixations on scene representation. Perception & Psychophysics 68: 759–769. 17076344 10.3758/bf03193699

[bibr56-2041669517724808] Intraub H. Richardson M. (1989) Wide-angle memories of close-up scenes. Journal of Experimental Psychology: Learning, Memory, and Cognition 15: 179. 10.1037//0278-7393.15.2.179 2522508

[bibr57-2041669517724808] James T. W. Huh E. Kim S. (2010) Temporal and spatial integration of face, object, and scene features in occipito-temporal cortex. Brain and Cognition 74: 112–122. 20727652 10.1016/j.bandc.2010.07.007

[bibr58-2041669517724808] Janik S. W. Wellens A. R. Goldberg M. L. Dell’Osso L. F. (1978) Eyes as the center of focus in the visual examination of human faces. Perceptual and Motor Skills 47: 857–858. 740480 10.2466/pms.1978.47.3.857

[bibr59-2041669517724808] Johansson R. Holsanova J. Holmqvist K. (2011) The dispersion of eye movements during visual imagery is related to individual differences in spatial imagery ability. In: Carlson L. Hölscher C. Shipley T. (eds) Expanding the space of cognitive science: Proceedings of the 33rd Annual Meeting of the Cognitive Science Society, Austin, TX: Cognitive Science Society, pp. 1200–1205.

[bibr60-2041669517724808] Johnson M. K. Hashtroudi S. Lindsay D. S. (1993) Source monitoring. Psychological Bulletin 114: 3. 8346328 10.1037/0033-2909.114.1.3

[bibr61-2041669517724808] Kang S. M. Shaver P. R. (2004) Individual differences in emotional complexity: Their psychological implications. Journal of Personality 72: 687–726. Retrieved from 10.1111/j.0022-3506.2004.00277.x. 15210014

[bibr62-2041669517724808] Kanizsa, G. (1979). *Organization in Vision: Essays on Geslalt Perception*. New York: Praeger.

[bibr63-2041669517724808] Kanwisher N. (2001) Faces and places: Of central (and peripheral) interest. Nature Neuroscience 4: 455–456. 11319548 10.1038/87399

[bibr64-2041669517724808] Kanwisher N. McDermott J. Chun M. M. (1997) The fusiform face area: A module in human extrastriate cortex specialized for face perception. The Journal of Neuroscience 17: 4302–4311. 9151747 10.1523/JNEUROSCI.17-11-04302.1997 PMC6573547

[bibr65-2041669517724808] Kim S. Dede A. J. Hopkins R. O. Squire L. R. (2015) Memory, scene construction, and the human hippocampus. Proceedings of the National Academy of Sciences 112: 4767–4772. 10.1073/pnas.1503863112 PMC4403152 25825712

[bibr66-2041669517724808] Klin A. Jones W. Schultz R. Volkmar F. (2003) The enactive mind, or from actions to cognition: Lessons from autism. Philosophical Transactions of the Royal Society of London B: Biological Sciences 358: 345–360. 12639332 10.1098/rstb.2002.1202 PMC1693114

[bibr67-2041669517724808] Komatsu H. (2006) The neural mechanisms of perceptual filling-in. Nature Reviews Neuroscience 7: 220–231. 16495943 10.1038/nrn1869

[bibr68-2041669517724808] Kosslyn S. M. Koenig O. (1992) Wet mind: The new cognitive neuroscience, New York, NY: Free Press.

[bibr69-2041669517724808] Kosslyn S. M. Pascual-Leone A. Felician O. Camposano S. Keenan J. P. Ganis G. Alpert N. M. (1999) The role of area 17 in visual imagery: Convergent evidence from PET and rTMS. Science 284: 167–170. 10102821 10.1126/science.284.5411.167

[bibr70-2041669517724808] Kozhevnikov, M., & Blazhenkova, O. (2013). Individual differences in object versus spatial imagery: from neural correlates to real-world applications. In S. Lacey & R. Lawson (Eds.), *Multisensory Imagery* (pp. 299–318). New York: Springer Publishing Company.

[bibr71-2041669517724808] Kozhevnikov M. Kosslyn S. Shephard J. (2005) Spatial versus object visualizers: A new characterization of visual cognitive style. Memory & Cognition 33: 710–726. 16248335 10.3758/bf03195337

[bibr72-2041669517724808] Laeng B. Teodorescu D. S. (2002) Eye scanpaths during visual imagery reenact those of perception of the same visual scene. Cognitive Science 26: 207–231.

[bibr73-2041669517724808] Landis T. Cummings J. L. Benson D. F. Palmer E. P. (1986) Loss of topographic familiarity: An environmental agnosia. Archives of Neurology 43: 132–136. 3947250 10.1001/archneur.1986.00520020026011

[bibr74-2041669517724808] Levy I. Hasson U. Avidan G. Hendler T. Malach R. (2001) Center–periphery organization of human object areas. Nature Neuroscience 4: 533–539. 11319563 10.1038/87490

[bibr75-2041669517724808] Loftus E. F. (2003) Make-believe memories. American Psychologist 58: 867. 10.1037/0003-066X.58.11.867 14609374

[bibr76-2041669517724808] Lundqvist D. Flykt A. Öhman A. (1998) The Karolinska directed emotional faces—KDEF, Stockholm, Sweden: Department of Clinical Neuroscience, Psychology Section, Karolinska Institutet.

[bibr77-2041669517724808] Markham R. Hynes L. (1993) The effect of vividness of imagery on reality monitoring. Journal of Mental Imagery 17: 159–170.

[bibr78-2041669517724808] Marks D. F. (1973) Visual imagery differences in the recall of pictures. British Journal of Psychology 64: 17–24. 4742442 10.1111/j.2044-8295.1973.tb01322.x

[bibr79-2041669517724808] Mast F. W. Kosslyn S. M. (2002) Eye movements during visual mental imagery. Trends in Cognitive Sciences 6: 271–272. 12110350 10.1016/s1364-6613(02)01931-9

[bibr80-2041669517724808] Mathews A. Mackintosh B. (2004) Take a closer look: Emotion modifies the boundary extension effect. Emotion 4: 36. 15053725 10.1037/1528-3542.4.1.36

[bibr81-2041669517724808] Maurer D. Le Grand R. Mondloch C. J. (2002) The many faces of configural processing. Trends in Cognitive Sciences 6: 255–260. 12039607 10.1016/s1364-6613(02)01903-4

[bibr82-2041669517724808] Mazard A. Tzourio-Mazoyer N. Crivello F. Mazoyer B. Mellet E. (2004) A PET meta-analysis of object and spatial mental imagery. European Journal of Cognitive Psychology 16: 673–695.

[bibr83-2041669517724808] Mazzoni G. Memon A. (2003) Imagination can create false autobiographical memories. Psychological Science 14: 186–188. 12661683 10.1046/j.1432-1327.1999.00020.x

[bibr84-2041669517724808] McCarthy G. Puce A. Gore J. C. Allison T. (1997) Face-specific processing in the human fusiform gyrus. Journal of Cognitive Neuroscience 9: 605–610. 23965119 10.1162/jocn.1997.9.5.605

[bibr85-2041669517724808] McDunn B. A. Siddiqui A. P. Brown J. M. (2014) Seeking the boundary of boundary extension. Psychonomic Bulletin & Review 21: 370–375. 23921509 10.3758/s13423-013-0494-0

[bibr86-2041669517724808] McKelvie S. J. (1976) The role of eyes and mouth in the memory of a face. American Journal of Psychology 9: 311–323.

[bibr87-2041669517724808] Mechelli A. Price C. J. Friston K. J. Ishai A. (2004) Where bottom-up meets top-down: Neuronal interactions during perception and imagery. Cerebral Cortex 14: 1256–1265. 15192010 10.1093/cercor/bhh087

[bibr88-2041669517724808] Ménétrier E. Didierjean A. Vieillard S. (2013) Is boundary extension emotionally selective? The Quarterly Journal of Experimental Psychology 66: 635–641. 23445174 10.1080/17470218.2013.764332

[bibr89-2041669517724808] Mertens I. Siegmund H. Grüsser O. J. (1993) Gaze motor asymmetries in the perception of faces during a memory task. Neuropsychologia 31: 989–998. 8232855 10.1016/0028-3932(93)90154-r

[bibr90-2041669517724808] Mullally S. L. Intraub H. Maguire E. A. (2012) Attenuated boundary extension produces a paradoxical memory advantage in amnesic patients. Current Biology 22: 261–268. 22264610 10.1016/j.cub.2012.01.001 PMC3315012

[bibr91-2041669517724808] Munger M. Owens T. R. Conway J. (2005) Are boundary extension and representational momentum related? Visual Cognition 12: 1041–1056.

[bibr92-2041669517724808] Munger M. P. Multhaup K. S. (2016) No imagination effect on boundary extension. Memory & Cognition 44: 73–88. 26250804 10.3758/s13421-015-0541-3

[bibr93-2041669517724808] O'Craven K. M. Kanwisher N. (2000) Mental imagery of faces and places activates corresponding stimulus-specific brain regions. Journal of Cognitive Neuroscience 12: 1013–1023. 11177421 10.1162/08989290051137549

[bibr94-2041669517724808] Park S. Intraub H. Yi D. J. Widders D. Chun M. M. (2007) Beyond the edges of a view: Boundary extension in human scene-selective visual cortex. Neuron 54: 335–342. 17442252 10.1016/j.neuron.2007.04.006

[bibr96-2041669517724808] Pashler H. (1994) Dual-task interference in simple tasks: Data and theory. Psychological Bulletin 116: 220. 7972591 10.1037/0033-2909.116.2.220

[bibr97-2041669517724808] Pellicano E. Rhodes G. Peters M. (2006) Are preschoolers sensitive to configural information in faces? Developmental Science 9: 270–277. 16669797 10.1111/j.1467-7687.2006.00489.x

[bibr98-2041669517724808] Peterson, D. (2016). *Digital photo secrets. Cropping 101*. Retrieved from http://www.digital-photo-secrets.com/tip/3497/cropping-101/.

[bibr99-2041669517724808] Porter S. Spencer L. Birt A. R. (2003) Blinded by emotion? Effect of the emotionality of a scene on susceptibility to false memories. Canadian Journal of Behavioural Science/Revue canadienne des sciences du comportement 35: 165.

[bibr100-2041669517724808] Quinn P. C. Intraub H. (2007) Perceiving “outside the box” occurs early in development: Evidence for boundary extension in three-to seven-month-old infants. Child Development 78: 324–334. 17328708 10.1111/j.1467-8624.2007.01000.x

[bibr101-2041669517724808] Ramachandran V. S. Gregory R. L. (1991) Perceptual filling in of artificially induced scotomas in human vision. Nature 350: 699–702. 2023631 10.1038/350699a0

[bibr102-2041669517724808] Roediger H. L. III (1996) Memory illusions. Journal of Memory and Language 35: 76–100.

[bibr951-2041669517724808] Russell, J. A. (1980). A circumplex model of affect. *Journal of Personality and Social Psychology*, *39*, 1161–1178. 10.1037//0022-3514.79.2.286 10948981

[bibr103-2041669517724808] Safer M. A. Christianson S. Å. Autry M. W. Österlund K. (1998) Tunnel memory for traumatic events. Applied Cognitive Psychology 12: 99–117.

[bibr104-2041669517724808] Schlegel K. Grandjean D. Scherer K. R. (2014) Introducing the Geneva Emotion Recognition Test: An example of Rasch-based test development. Psychological Assessment 26: 666. 24295238 10.1037/a0035246

[bibr105-2041669517724808] Seamon J. G. Schlegel S. E. Hiester P. M. Landau S. M. Blumenthal B. F. (2002) Misremembering pictured objects: People of all ages demonstrate the boundary extension illusion. The American Journal of Psychology 115: 151–167. 12041005

[bibr106-2041669517724808] Shepard R. N. Metzler J. (1971) Mental rotation of three-dimensional objects. Science 171: 701–703. 5540314 10.1126/science.171.3972.701

[bibr107-2041669517724808] Shipley T. F. Kellman P. J. (1992) Perception of partly occluded objects and illusory figures: Evidence for an identity hypothesis. Journal of Experimental Psychology: Human Perception and Performance 18: 106.

[bibr108-2041669517724808] Tanaka J. W. Farah M. J. (1993) Parts and wholes in face recognition. The Quarterly Journal of Experimental Psychology 46: 225–245. 8316637 10.1080/14640749308401045

[bibr109-2041669517724808] Tanaka J. W. Sengco J. A. (1997) Features and their configuration in face recognition. Memory & Cognition 25: 583–592. 9337578 10.3758/bf03211301

[bibr110-2041669517724808] Taylor K. J. Henson R. N. Graham K. S. (2007) Recognition memory for faces and scenes in amnesia: Dissociable roles of medial temporal lobe structures. Neuropsychologia 45: 2428–2438. 17509626 10.1016/j.neuropsychologia.2007.04.004

[bibr111-2041669517724808] Thomas A. K. Bulevich J. B. Loftus E. F. (2003) Exploring the role of repetition and sensory elaboration in the imagination inflation effect. Memory & Cognition 31: 630–640. 12872878 10.3758/bf03196103

[bibr112-2041669517724808] Ungerleider L. G. Haxby J. V. (1994) ‘What’ and ‘where’ in the human brain. Current Opinion in Neurobiology 4: 157–165. 8038571 10.1016/0959-4388(94)90066-3

[bibr113-2041669517724808] Vannucci M. Pelagatti C. Chiorri C. Mazzoni G. (2016) Visual object imagery and autobiographical memory: Object imagers are better at remembering their personal past. Memory 24: 455–470. 25751732 10.1080/09658211.2015.1018277

[bibr114-2041669517724808] Walker-Smith G. J. Gale A. G. Findlay L. M. (1977) Eye movement strategies involved in face perception. Perception 6: 313–326. 866088 10.1068/p060313

[bibr115-2041669517724808] Walls G. L. (1954) The filling-in process. American Journal of Optometry and Archives of American Academy of Optometry 31: 329–341. 13180658 10.1097/00006324-195407000-00001

[bibr116-2041669517724808] Wells G. L. Small M. Penrod S. Malpass R. S. Fulero S. M. Brimacombe C. E. (1998) Eyewitness identification procedures: Recommendations for lineups and photospreads. Law and Human Behavior 22: 603.

[bibr117-2041669517724808] Yantis S. (1995) Perceived continuity of occluded visual objects. Psychological Science 6: 182–186.

[bibr118-2041669517724808] Yarbus A. L. (1967) Eye movements and vision, New York, NY: Plenum.

[bibr119-2041669517724808] Zeidman P. Mullally S. L. Maguire E. A. (2014) Constructing, perceiving, and maintaining scenes: Hippocampal activity and connectivity. Cerebral Cortex 25: 3836–3855. 25405941 10.1093/cercor/bhu266 PMC4585517

